# *SLC30A4‐AS1* Mediates the Senescence of Periodontal Ligament Stem Cells in Inflammatory Environments via the Alternative Splicing of *TP53BP1*

**DOI:** 10.1111/cpr.13778

**Published:** 2024-11-21

**Authors:** Mei Xu, Dian Gan, Xi‐Yu Zhang, Xiao‐Tao He, Rui Xin Wu, Yuan Yin, Rui Jin, Lin Li, Yu‐Jie Tan, Fa‐Ming Chen, Xuan Li, Bei‐Min Tian

**Affiliations:** ^1^ State Key Laboratory of Oral & Maxillofacial Reconstruction and Regeneration, National Clinical Research Center for Oral Diseases, Shaanxi International Joint Research Center for Oral Diseases, Department of Periodontology, School of Stomatology The Fourth Military Medical University Xi'an China

**Keywords:** alternative splicing, cell senescence, inflammation, periodontal ligament stem cells, TP53BP1

## Abstract

Periodontal ligament stem cells (PDLSCs) are key cells that suppress periodontal damage during both the progression and recovery stages of periodontitis. Although substantial evidence has demonstrated that incubation under an inflammatory condition may accelerate senescence of PDLSCs, whether cellular senescence in response to inflammatory incubation contributes to cell dysfunction remain unexplored. In this study, we first observed inflammation‐caused PDLSC senescence in periodontitis based on comparisons of matched patients, and this cellular senescence was demonstrated in healthy cells that were subjected to inflammatory conditions. We subsequently designed further experiments to investigate the possible mechanism underlying inflammation‐induced PDLSC senescence with a particular focus on the role of long noncoding RNAs (lncRNAs). LncRNA microarray analysis and functional gain/loss studies revealed *SLC30A4‐AS1* as a regulator of inflammation‐mediated PDLSC senescence. By full‐length transcriptome sequencing, we found that *SLC30A4‐AS1* interacted with SRSF3 to affect the alternative splicing (AS) of *TP53BP1* and alter the expression of *TP53BP1*‐204. Further functional studies showed that decreased expression of *TP53BP1*‐204 reversed PDLSC senescence, and *SLC30A4‐AS1* overexpression‐induced PDLSC senescence was abolished by *TP53BP1*‐204 knockdown. Our data suggest for the first time that *SLC30A4‐AS1* plays a key role in regulating PDLSC senescence in inflammatory environments by modulating the AS of *TP53BP1*.

## Graphical abstract

Briefly, *SLC30A4‐AS1* is upregulated in senescent PDLSCs mediated by inflammation. *SLC30A4‐AS1* binds the splicing factor SRSF3 to affect the alternative splicing of *TP53BP1* and alter the expression of its transcript *TP53BP1*‐204. Knockdown of *TP53BP1*‐204 expression can improve the senescence of PDLSCs and improve the osteogenic differentiation ability of PDLSCs.

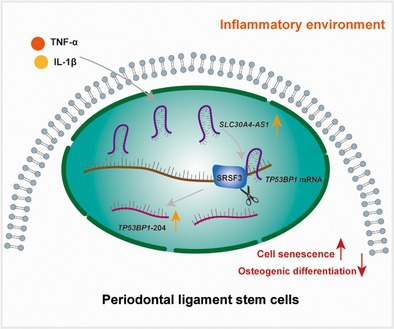

## 1 Introduction

Periodontitis has a high incidence and causes great harm, and it seriously affects human oral health. Destruction of periodontal tissues due to periodontitis is the main cause of tooth loss in adults [1]. Therefore, the goals of the clinical treatment of periodontal disease include controlling the development of periodontal disease and repairing periodontal tissue defects. Extracted from periodontal ligaments, periodontal ligament stem cells (PDLSCs) possess the ability to differentiate into osteogenic and adipogenic cells within a controlled environment in vitro, thereby building molecularly comparable structures of periodontal ligament tissue in vivo [2]. The PDLSC transplantation has good prospects for application in clinical practice [3, 4]. Thus, correctly understanding the regenerative ability of PDLSCs is highly important for promoting periodontal tissue repair.

During the utilisation of stem cell therapy in correcting periodontal defects, the significant restoration capability demonstrated by PDLSCs is pivotal in reimagining periodontal structures. At present, PDLSCs are derived mainly from teeth that are extracted because they become impacted or for orthodontic reasons [5]. However, these resources are not always available, and it is difficult to obtain large quantities of PDLSCs for clinical research due to limitations such as operating techniques [6, 7, 8]. Recently, numerous studies affirm that PDLSCs can be isolated from the inflamed periodontal ligament tissues in individuals afflicted with periodontitis (P‐PDLSCs), suggesting this tissue as a more readily obtainable alternative to healthy periodontal ligament tissues [9]. P‐PDLSCs are more difficult to establish than PDLSCs from non‐periodontitis patients, and the slow cell proliferation and poor cell status of P‐PDLSCs make it difficult to large‐scale culture to meet the requirements of multiple experiments. In addition, whether P‐PDLSCs can fully participate in clinical treatment depends on whether P‐PDLSCs have good activity and normal function. Analysis of the literature suggests that some functions of P‐PDLSCs are impaired compared to those of healthy PDLSCs [8, 10].

Similar to adult cells, stem cells undergo senescence. The main cause of non‐age‐related tissue stem cell senescence is changes in the microenvironment surrounding the stem cells. The prevailing feature of chronic inflammation within periodontitis lies in its capacity to induce pathological alterations within affected tissues that subsequently foster an accommodative ecological niche for the onset of cellular senescence [11]. Studies have shown that cell senescence is an important factor that affects cell activity and function and reduces the effectiveness of cell therapy [12]. The PDLSC senescence that is induced by inflammatory conditions may be an important cause of impaired P‐PDLSC regeneration. Delaying cell senescence can not only be used as a treatment strategy for aging‐related diseases but also provide better sources of cells for cell therapy. However, the molecular regulatory network that is involved in P‐PDLSC senescence is not fully understood, and safe and effective molecular targets are lacking.

Alternative splicing (AS) refers to the splicing and synthesis of transcripts from pre‐mRNAs to generate mature mRNAs, and this process enables the genome to produce highly diverse proteins from a single RNA sequence. In humans, 95% of genes undergo splicing [13]. Some anomalies in RNA functionality are correlated with age‐induced pathologies, where AS holds a pivotal role in orchestrating cellular senescence implicated in tissue and organ deterioration [14, 15]. Long noncoding RNAs (lncRNAs) are more than 200 nucleotides in length, and are a class of non‐protein‐synthesising RNA molecules [16]. LncRNAs have characteristics that are similar to those of mRNAs, such as posttranscriptional modification and splicing, and lncRNAs play roles in aging‐related cellular mechanisms. LncRNAs serve as pivotal regulators of cellular proliferation, differentiation, apoptosis, senescence, and a variety of other essential physiological functions [17, 18, 19]. However, compared to those of coding RNAs, the functions of lncRNAs are far from fully understood. On the one hand, lncRNAs, which are products of pre‐mRNAs, are involved in the regulation of the aging process. On the other hand, the AS of precursor lncRNAs results in the production of aging‐associated aberrant splice variants. Moreover, long non‐coding RNAs either directly or indirectly orchestrate AS events within downstream effector genes, consequently impacting the initiation and progression of aging phenomena [20, 21].

We hypothesized that the inflammatory microenvironment in periodontitis promotes the senescence of PDLSCs, that senescence affects the expression of lncRNAs in PDLSCs, and that lncRNAs regulate the abnormal AS of senescence‐related proteins and ultimately affect the regenerative function of PDLSCs. P‐PDLSCs were obtained from clinical sources, and PDLSCs were stimulated with inflammatory factors to mimic the inflammatory microenvironment in order to elucidate the effect of inflammation on PDLSC senescence. Mechanistically, key lncRNAs that are involved in inflammation‐mediated PDLSC senescence were screened and identified. In addition, through the use of full‐length transcriptome sequencing, we explored how lncRNAs mediate the occurrence of abnormal AS events. This project provides new ideas for elucidating the pathogenesis of periodontal disease, lays a theoretical foundation for guiding the quality control and modification of PDLSCs for clinical use, and provides research experience for promoting the ideal therapeutic effects of PDLSCs that are used in clinical treatment.

## 2 Materials and Methods

### 2.1 Isolation and Characterisation of PDLSCs

PDLSCs were isolated from 10 patients (30–50 years old) with periodontitis who had no systemic disease as previously described [22], each patient had at least one tooth that was severely affected by periodontitis that needed to be extracted as well as one healthy blocked or non‐functioning tooth that needed to be extracted. Each patient's diagnosis was reviewed and approved by at least two physicians. All the donors provided signed informed consent and agreed to provide their extracted teeth for research purposes. Briefly, PDL tissues were delicately detached from the dental structure via scraping and subsequently catalytically digested with type I collagenase (3 mg/mL) (DIYIBio, China). The tissues were then incubated in α‐MEM (Gibco, USA) supplemented with 10% fetal bovine serum (FBS; Sijiqing, China), penicillin and streptomycin (100 U/mL) (Invitrogen, USA). Finally, a meticulous limited dilution methodology was employed to isolate populations of PDLSCs, specifically cells derived from the 2nd to 5th passages for ensuing investigations.

### 2.2 Cell Treatments

To generate I‐PDLSCs, an inflammatory environment was established using complete medium supplemented with TNF‐α (10 ng/mL) and IL‐1β (10 ng/mL) (Novoprotein, China). To determine how the presence of inflammatory cytokines affects cellular senescence, I‐PDLSCs were cultured in this inflammatory environment for 7 days before physiological function was analysed.

### 2.3 Colony‐Formation Unit Assay

PDLSCs were digested and centrifuged. Then, the suspensions were plated in 6‐mm dish at a density of 100 cells, the medium was changed every 3 days. After culture for 14 days, the cells were incubated in an 1% crystal violet staining solution (Beyotime, China) for 15 min. Upon thorough PBS wash, observation was conducted under an optical microscope. The colony formation ability of the cells was analysed by determining the number and size of the colonies.

### 2.4 CCK‐8 Assay

PDLSCs were seeded in 96‐well plates at a density of 3 × 10^4^/well. A Cell Counting Kit‐8 (CCK‐8) assay was performed after 1–8 days of culture. Briefly, adding mixture containing 10 μL CCK‐8 reagent (Beyotime, China) and 90 μL medium to each well, the plates were incubated for 2 h at 37°C, subsequent to which the OD value had been evaluated at the wavelength of 450 nm using an automated plate reader apparatus.

### 2.5 Flow Cytometry

PDLSCs were digested and centrifuged to obtain single‐cell suspensions. The 500 μL suspensions containing 10^6^ cells were incubated with antibodies at 4°C for 30 min in the dark. The antibodies that were used are shown in Table S2. After washing with PBS, the samples were centrifuged, resuspended in 500 μL of culture medium, and assayed using flow cytometry. The apoptosis of cells was analysed using an Annexin V‐FITC/propidium iodide (PI) apoptosis detection kit (Yeasen Biotechnology, China), according to our previously reported methods [23]. The 200 μL single‐cell suspensions containing 10^6^ cells and 5 μL of Annexin V‐FITC and 5 μL of PI for 15 min at room temperature. After washing with PBS, cells were subjected to flow cytometry.

### 2.6 Osteogenic Differentiation Assay

PDLSCs were plated at a density of 1 × 10^5^/well in six‐well plates pre‐coated with 0.1% gelatin. The medium was replaced with osteogenic medium when cell confluences reached 60%–70% (Cyagen, China). The fresh osteogenic medium was changed every 3 days. ALP staining and quantitative assays (Beyotime, China) were performed after 14 days of culture according to the manufacturer's instructions. Alizarin red staining (Beyotime, China) was performed after 21 days. To quantitatively analyse the mineralization nodule formation, 200 μL of 2% cetylpyridine chloride was added to each well, and the samples were incubated for 2 h at room temperature. The solution was transferred to a separate 96‐well plate, and the OD values were measured at a wavelength of 562 nm.

### 2.7 Adipogenic Differentiation Assay

PDLSCs were seeded at a density of 1 × 10^5^ cells/well in six‐well plates pre‐coated with 0.1% gelatin. When the cells reached 80%–90% confluence, the medium was replaced by lipid induction medium (Cyagen, China), which was changed every 3 days. Half fluid exchanges were observed every 2 days after lipid droplet formation. Oil Red O staining was performed according to the manufacturer's instructions after 14 days of culture.

### 2.8 Chondrogenic Differentiation Assay

The cells were resuspended in chondrogenic induction medium (Cyagen, China) and seeded in six‐well plates at a density of 1×10^5^ cells/well. The medium was changed every 3 days, and (1%) toluidine blue staining was performed according to the manufacturer's instructions after 3 weeks of culture.

### 2.9 ELISA

The cell supernatant was collected and centrifuged to remove cell debris. The amounts of MCP‐1, IL‐8, IL‐6, and IFN‐γ that were secreted into the supernatants of different groups were measured with corresponding ELISA kits (Neobioscience, China) according to the manufacturer's protocol, and the OD values were measured with a microplate reader.

### 2.10 qRT–PCR

Total RNA was isolated from cells using TRIzol reagent (Sigma–Aldrich, USA). RNA was converted to cDNA using Hifair Strand cDNA Synthesis SuperMix (Yeasen, China) according to the manufacturer's instructions. qRT–PCR was performed using Hieff qPCR SYBR Green Master Mix (Yeasen, China) to measured gene expression levels. The expression of relative mRNA was quantified using the 2^−ΔΔCt^ method. The sequences of the primers that were used are shown in Table S1.

### 2.11 Western Blotting

The total protein of PDLSCs was extracted with RIPA buffer (Beyotime, China). After the protein concentrations were determined by the Bradford method, protein was separated via SDS–polyacrylamide gel electrophoresis and subsequently transferred to PVDF membranes. The membranes were then blocked with 5% bovine serum albumin (BSA, Sigma–Aldrich, USA) solution for 1 h at room temperature, and incubated with primary antibodies (P53, P27, P21, P16, and GAPDH) overnight at 4°C. After washing with TBST, the membranes were incubated with secondary antibodies for 1 h at room temperature and visualised using a chemiluminescence imaging system. The antibodies that were used are shown in Table S2.

### 2.12 SA‐β‐Gal Staining

After 7 days of culture, the cells were fixed at room temperature for 30 min. After washing with PBS, the staining solution (Solarbio, China) was added, and the cells were incubated at 37°C overnight without using 5% CO_2_ cell incubator. The staining solution was removed the next day, and the cells were washed with PBS and observed under a microscope.

### 2.13 Immunofluorescence

Subsequent to fixation of the cells with a 4% paraformaldehyde solution for 15 min, they were permeabilized with 0.1% Triton X‐100 solution for 30 min. Post‐permeabilization, the cells were blocked with 3% BSA for an hour. Subsequently, the cells were incubated with primary antibodies targeted against γ‐H2AX at 4°C overnight. These cells were then washed thoroughly with PBS and subsequently incubated with fluorescently labelled secondary antibodies for an hour in darkness. DAPI served as a marker for nuclear staining. Fluorescence images were acquired using an Olympus FV1000 confocal laser scanning microscope and analysed using FV10‐ASW4.2 software. The antibodies utilised for this experiment are listed in Table S2.

### 2.14 LncRNA Microarray Analysis

After 7 days of culture, 1 mL of TRIzol reagent was added directly to the dish containing 2 × 10^6^ H‐PDLSCs or I‐PDLSCs after discarding the culture medium. After several repeated cycles of aspiration and resuspension, it could be seen that the cell layer was completely lysed. All the solutions were collected in RNase‐free 1.5‐mL EP tubes, stored at −80°C and subsequently sent to Aksomics Co. Ltd. for analysis by Arraystar Microarray (more than 1 × 10^6^ cells were used to prepare each sample). Before the RNA was labelled, an Agilent ND‐1000 was used to determine whether the RNA was degraded and to determine the RNA concentration. An Arraystar RNA Flash Labeling Kit was used to label the samples. An Agilent SureHyb was used to perform the hybridization assay, and the microarrays were washed and scanned with an Agilent RNA Microarray Scanner. The chip probe signal values were collected using Agilent Feature Extraction software. Chip quantile standardisation was performed using Agilent GeneSpring GX version 12.1 software. Differentially expressed lncRNAs were selected using Agilent GeneSpring GX version 12.1 software.

### 2.15 Cytoplasmic/Nuclear Fraction Isolation

Nuclear and cytoplasmic fractions were extracted from PDLSCs using a Nuclear and Cytoplasmic Protein Extraction Kit (Beyotime, China) according to the manufacturer's instructions. RNA was subsequently extracted, and mRNA expression was determined. LncRNA expression levels as well as nuclear reference (U6) and cytoplasmic reference (β‐actin) levels in each fraction were normalised to their levels in whole‐cell RNA samples, which were set to 100% [24].

### 2.16 Fluorescence In Situ Hybridization (FISH)

Reagents for the experiments were purchased from Servicebio technology, and the probe sequences used in the experiments were as follows: 5′‐ACAGAGTGAGATCAG GATCATTGGCATC‐3′, 5′‐AGTCTATTCCATCCTCTTTTTGCTTCTT‐3′, 5′‐GCTG TACATTATAACAGTGATTCTCGAAGTG‐3′. Samples were fixed in hybridization fixative for 20 min and washed in PBS. Proteinase K (5 μg/mL) was dropped and digested at 37°C for 5 min. Prehybridization solution was added dropfold and incubated for 1 h at 37°C. Probe containing hybridization solution was added dropingly and hybridised overnight at 40°C. After hybridization, the samples were washed and hybridised dropfold with the corresponding branching probe at 40°C for 45 min. They were washed after hybridization. The corresponding signal probe was added dropwise at a dilution ratio of 1:200. The cells were incubated at 42°C for 3 h. After washing, the cells were blocked with 3% BSA for 30 min. Primary antibodies were incubated overnight at 4°C. The secondary antibodies were incubated for 50 min at room temperature. DAPI was used to label the nuclei, and after washing, anti‐fluorescence quenching sealing agent was dropped to seal the tablets. The cells were observed and the images were collected under a Nikon upright fluorescence microscope and analysed using CaseViewer2.3 software.

### 2.17 Transfections

The cells were plated in cell culture plates 1 day in advance, the cell confluence was 60%–80% at the time of transfection, and transfection was carried out when the cells were in good condition. The siRNAs or antisense oligonucleotides (ASOs) that were to silence target lncRNAs were purchased from TSINGKE, and their sequences are shown in Table S3. PDLSCs were transfected using Transfecter Cell Reagent (Micropoly, China). Briefly, 20 μM si/ASO‐NC or ASO/siRNA was first mixed with transfection reagent. The mixture was incubated at room temperature for 10 min, followed by dilution with medium according to manufacturer's instructions. Then, the reagents were added to cells and incubated in an incubator. The silencing efficiency was measured after 48 h.

The lentiviral vector pLVX‐IRES‐Puro containing target lncRNA sequences was purchased from TSINGKE. A total of 2 × 10^5^ PDLSCs were cultured 1 day in advance, and the cells were subsequently transfected with lentivirus (MOI = 10) for 24 h. Subsequently, the medium was replaced with fresh culture medium. PDLSCs that survived after screening with 5 μg/mL puromycin (DIYIBio, China) for 48 h were cultured. The overexpression efficiencies of the target lncRNAs were measured after passaging.

### 2.18 Full‐Length Transcriptome Sequencing

The number of cells required for one RNA extraction reaction was 3 × 10^6^ ~ 1 × 10^7^ cells. The cells were seeded in 10‐cm cell culture dishes at a density of 2 × 10^5^/dish and then cultured for 7 days. The cells were lysed using TRIzol reagent, and all the samples were collected and added to RNase‐free 1.5‐mL EP tubes. Then, the samples were sent to BioMaker Biotechnology Co. Ltd. for three‐generation ONT full‐length transcriptome sequencing data analysis. The experimental methodology was implemented in accordance with the established protocol furnished by Oxford Nanopore Technologies. The full‐length sequences were aligned with the reference genome using minimap2 software, and the aligned sequences were clustered using pinfish software. The transcripts that were identified by full‐length sequencing were compared with known transcripts of the genome using Gffcompare, and new genes and transcripts were found to complement the genome annotation. The variable splice types present in each sample were identified by Astalavista software, and counts per million (CPM) was used as a measure of transcript or gene expression levels.

### 2.19 Dual‐Luciferase Reporter Assay

Cells were plated in 96‐well plates, permitting a 24‐h incubation to achieve a cellular density approximating 70~80% confluence. Afterward, 0.15 μg of reporter gene vector and 0.45 μL of Hieff Trans reagent were diluted with 10 μL of medium without FBS per well. Afterward, these solutions were mixed thoroughly to form the DNA‐Hieff Trans complex. Hieff Trans reagent (0.3 μL) was diluted with 3 μL of medium without FBS per well, 0.25 μL of lncRNA (20 μM) was added, and the solution was mixed thoroughly to form the RNA‐Hieff Trans complex. Eighty microliters of medium without FBS were added to each well of a 96‐well, the complex was added to the wells in a dropwise manner, and the cells were subsequently cultured in a 5% CO_2_ incubator at 37°C for 6 h. Then the medium was replaced with complete medium. The cells were lysed, and a working solution of luciferase firefly reaction and a solution of luciferase sea kidney reaction were prepared. Twenty microliters of cell lysate were added to a black enzyme plate. A volume of 100 μL firefly luciferase reaction solution or sea kidney luciferase reaction solution was added to the samples, after which the enzyme activity was measured after the samples had been incubated for 10 min. Assay ratio = firefly luciferase assay/kidney luciferase assay.

### 2.20 RNA Immunoprecipitation

Cells were collected and lysed by adding polysome lysis buffer containing protease inhibitor, and RNase inhibitor. The cell lysates were combined with DNase salt stock and DNase (20 U) and incubated at 37°C for 10 min. The samples were transferred to an ice bath. EDTA, EGTA, and DTT were quickly added at 4°C. The samples were centrifuged at 16,000 × g for 10 min, after which the supernatants were transferred to RNase‐free centrifuge tubes. The cell lysate samples were divided into triplicate aliquots as follows: IP: IgG: Input = 8:8:1. The IP and IgG samples were incubated with experimental antibodies and the same amount of IgG (negative control), respectively, at 4°C overnight. Subsequently, protein A/G beads was added and incubated for 1 h at 4°C. After washing three times, polysome washing buffer 1, DNase salt stock, and DNase (5 U) were added, and the beads were incubated at 37°C for 10 min. Then, the beads were collected via a magnetic rack, and the supernatants were removed. The beads from the IP and IgG samples were resuspended in polysome elution buffer, DTT, and proteinase K, while the beads from the Input samples were resuspended in DTT and proteinase K, and then, the beads were incubated for 1 h at 55°C. The RNA was eluted, and the magnetic beads were collected on a magnetic rack. Then, supernatants were transferred to new ribonuclease‐free centrifuge tubes for subsequent routine qRT‐PCR.

### 2.21 Animal Experiments

24 six‐week‐old male BALB/c nude mice were randomly divided into 4 groups (si‐NC + ov‐NC group, si‐NC + ov‐*SLC30A4‐AS1* group, si‐*TP53BP1‐*204 + ov‐NC group and si‐*TP53BP1‐*204 + ov‐*SLC30A4‐AS1* group, si‐NC + ov‐NC group as control group). PDLSC‐bone meal mixtures were prepared before the operation. One milliliter suspension containing 10^6^ cells was mixed with 30 mg Bio‐OSS (Geistlich Pharma, Switzerland) and incubated at 37°C for 6 h. The supernatant was discarded by centrifugation. Nude mice were prohibited from eating and drinking for 6 h before surgery and anaesthetised via intraperitoneal injection of 1% pentobarbital (0.1 mL/20 g). The dorsal skin was sterilised with iodophor, and an incision approximately 5 mm in length was made to bluntly detach the fascia to generate a subcutaneous pouch into which the PDLSC‐Bio‐OSS mixture was grafted. At 8 weeks after surgery, the nude mice were executed by using an overdose of anaesthetic (intraperitoneal injection 1 mL 1% pentobarbital), and the dorsal subcutaneous grafts were removed. Then, the grafts were fixed in 4% paraformaldehyde for 24 h. Bone tissue formation and markers of senescence were detected via HE staining, Masson staining and Immunohistochemistry.

### 2.22 HE, Masson Staining and Immunohistochemistry

After the implants were removed, they were fixed in 4% paraformaldehyde for 24 h and decalcified in 10% EDTA for 1 week. The implants were washed with running water for 2 days and paraffin embedded. The samples were cut into sections (5 μm), stained using an HE staining kit and Masson trichrome staining kit (Servicebio, China), sealed with neutral resin, and observed under a microscope. For immunohistochemistry analysis, the implants fixed in 4% polyformaldehyde, embedded in paraffin, sectioned, and stained with primary antibodies against P21 and γ‐H2AX. The images were collected under a Nikon upright fluorescence microscope and analysed using CaseViewer2.3 software.

### 2.23 Statistical Analysis

All the data in this study are presented as the means ± SDs of at least three independent experiments. GraphPad Prism 9 software was used for statistical analysis and graphing. When more than two groups of data were compared, one‐way difference analysis was used, followed by Tukey's multiple comparison test, Sidak's multiple comparison test, or Dunnett's multiple comparison test. Independent sample *t* tests were used for comparisons between two groups. A *p* value less than 0.05 was considered to indicate statistical significance.

## 3 Results

### 3.1 PDLSCs From Periodontitis Patients Exhibit Cellular Senescence

In this study, we acquired inflamed and healthy tooth samples from individuals who were diagnosed with periodontitis. Using these samples, we successfully established the P‐PDLSC and H‐PDLSC cell lines. These cells demonstrate the capability for proliferation and differentiation into distinct lineage pathways such as osteogenesis, adipogenesis, and chondrogenesis. This phenomenon has been validated via multiple investigative methodologies, including was confirmed through various experimental techniques, including a fibroblast colony‐forming unit assay (Figure S1A), CCK‐8 assay (Figure S1B), flow cytometry analysis (Figure S1C), and multilineage differentiation potential analysis (Figure S1D).

We cultured H‐PDLSCs and P‐PDLSCs simultaneously for 6 days. Then, P‐PDLSC senescence was systematically examined via a series of related experiments. We found that the proliferation of P‐PDLSCs was slower than that of H‐PDLSCs (Figure S2A), but there was no significant difference in apoptosis (Figure S2B). The senescence‐associated secretory phenotype (SASP) is characterised by the secretion of a series of inflammatory cytokines, chemokines, growth factors, and proteases by senescent cells [25]. Enzyme‐linked immunosorbent assays (ELISAs) demonstrated significant elevations in the release of MCP1, IL‐8, IL‐6, and IFN‐γ by P‐PDLSCs (Figure S2C). Quantitative real‐time polymerase chain reaction (qRT–PCR) revealed that the expression levels of senescence‐related genes (*P53*, *P27*, *P21*, and *P16*) were significantly increased in P‐PDLSCs (Figure S2D). Similarly, western blotting analysis revealed a significant increase in the levels of senescence‐related proteins (P53, P27, P21, and P16) in P‐PDLSCs (Figure S2E,F). Correspondingly, SA‐β‐gal activity, which reflects the degree of cellular senescence [26], was significantly increased in P‐PDLSCs (Figure S2G,H). The formation of discrete nuclear foci after γ‐H2AX phosphorylation strongly suggested that DNA double‐strand break had occurred [27]. Immunofluorescence assays showed that the expression of γ‐H2AX was significantly increased in P‐PDLSCs (Figure S2I,J). In addition, H‐PDLSCs and P‐PDLSCs were induced to undergo osteogenic differentiation for 28 days, and alizarin red staining and quantification confirmed that the extracellular mineralization of P‐PDLSCs was also reduced (Figure S2K,L). After induction, the expression levels of osteogenesis‐related genes (*ALP*, *OCN*, and *RUNX2*) in the P‐PDLSC group were significantly lower than those in the H‐PDLSC group (Figure S2M). These results indicated that P‐PDLSCs, which were derived from inflammatory tissues, exhibited more characteristics of senescence than H‐PDLSCs.

### 3.2 Inflammatory Cytokines Lead to PDLSC Senescence

**FIGURE 1:**
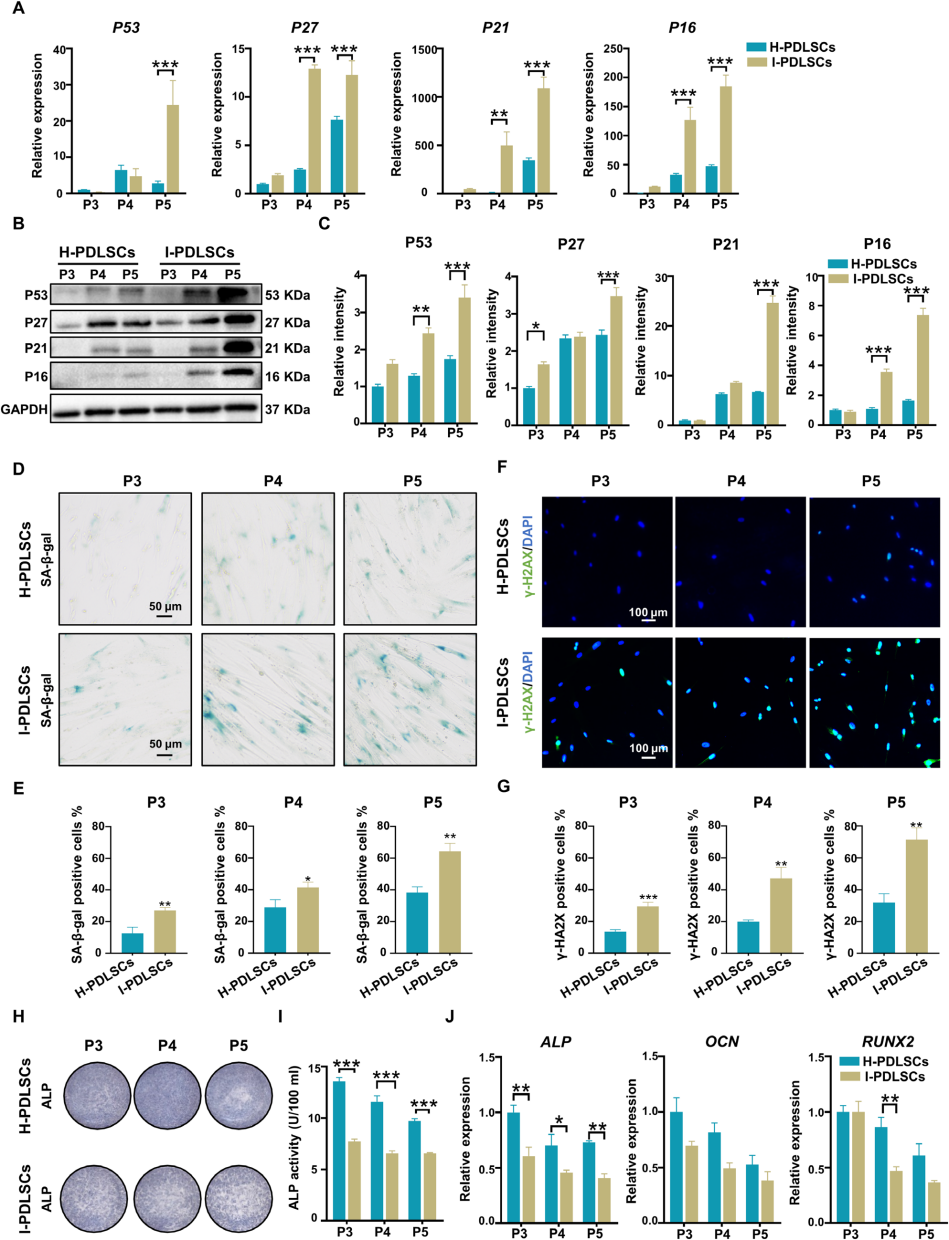
Stimulation with TNF‐α and IL‐1β led to PDLSC senescence. I‐PDLSCs were incubated in medium supplemented with IL‐1β and TNF‐α, and H‐PDLSCs were incubated in normal medium. (A) The expression levels of senescence‐related genes (*P53*, *P27*, *P21*, and *P16*) in PDLSCs were measured via qRT–PCR. (B) The expression of senescence‐related proteins (P53, P27, P21, and P16) was measured via western blotting. (C) Semiquantitative analysis of protein expression levels normalised to GAPDH expression levels. (D) Senescence‐associated β‐galactosidase in H‐PDLSCs and I‐PDLSCs was analysed via SA‐β‐gal staining in (scale bar: 50 μm). (E) Quantification of SA‐β‐Gal staining in H‐PDLSCs and I‐PDLSCs. (F) Immunofluorescence staining for γ‐H2AX in PDLSCs; nuclei were stained with DAPI (scale bar: 100 μm). (G) Percentage of γ‐H2AX‐positive H‐PDLSCs and I‐PDLSCs. (H) Representative image of ALP staining in PDLSCs after 14 days of osteogenic differentiation induction. (I) Quantification of ALP activity in PDLSCs after osteogenic induction for 14 days. (J) The expression levels of osteogenesis‐related genes (*ALP*, *OCN*, and *RUNX2*) in PDLSCs after osteogenic induction were measured via qRT–PCR. The data are presented as the means ± SDs (*n* = 3). *p < 0.05, **p < 0.01, and ***p < 0.001 represent significant differences between the indicated columns.

An inflammatory environment was established by adding 10 ng/mL TNF‐α and 10 ng/mL IL‐1β to the cell culture medium according to our previously established protocol [28], with slight modifications, and studies were preformed to determine how the presence of inflammatory cytokines affects the senescence of PDLSCs. In this study, H‐PDLSCs were incubated in normal environments and I‐PDLSCs were incubated in inflammatory environments. The PDLSCs from P3‐P5 were cultured for 6 days, after which the senescence of the PDLSCs in each group was evaluated. CCK‐8 analysis showed that the proliferation of I‐PDLSCs was significantly slower than that of H‐PDLSCs (Figure S3A), but no obvious difference in apoptosis was observed (Figure S3B). According to the qRT–PCR results, the expression levels of senescence‐related genes (*P53*, *P27*, *P21*, and *P16*) were significantly increased in I‐PDLSCs (Figure 1A). Similarly, incubating cells under inflammatory conditions significantly increased the levels of proteins (P53, P27, P21, and P16) (Figure 1B,C). Correspondingly, SA‐β‐gal activity was significantly increased in I‐PDLSCs (Figure 1D,E). Immunofluorescence assays showed that the expression of γ‐H2AX was significantly increased in I‐PDLSCs (Figure 1F,G). In addition, PDLSCs were induced to undergo osteogenic differentiation for 14 days. H‐PDLSCs exhibited increased tendency towards expressing positive alkaline phosphatase (ALP) staining (Figure 1H) and higher cellular ALP activity than I‐PDLSCs (Figure 1I). After induction, the expression levels of osteogenesis‐related genes (*ALP*, *OCN*, and *RUNX2*) in the I‐PDLSCs were significantly reduced compared to that measured in the H‐PDLSCs (Figure 1J). These results indicate that inflammatory factors can lead to the senescence of PDLSCs.

### 3.3 Identification of SLC30A4‐AS1 As a Key lncRNA That Is Related to PDLSC Senescence Under Inflammatory Conditions

**FIGURE 2:**
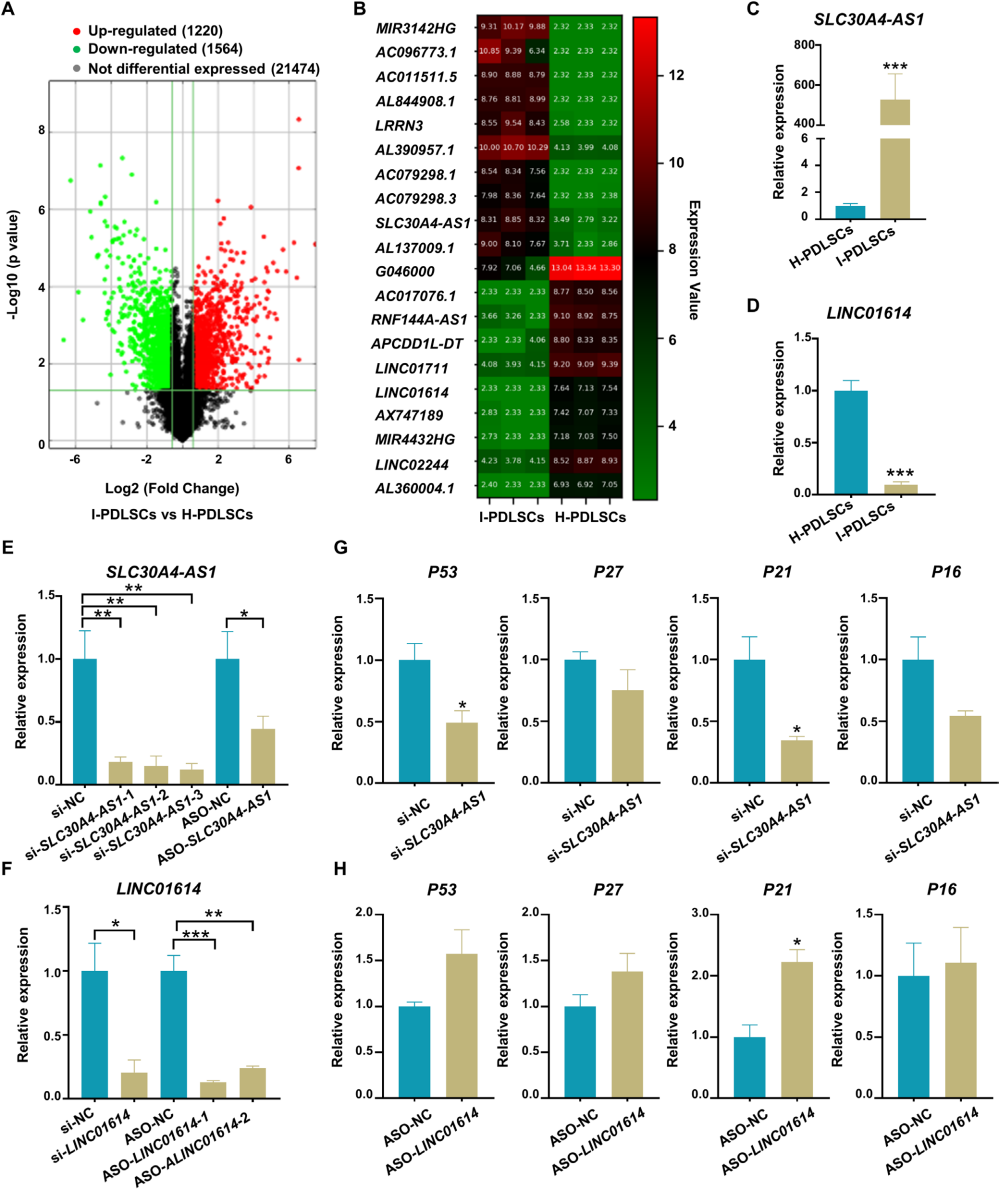
Identification and validation of *SLC30A4‐AS1* as a key lncRNA related to PDLSC senescence by microarray analysis. (A) Volcano plots of differentially expressed lncRNAs (fold change > 1.5 and adjusted *p* < 0.05) between H‐PDLSCs and I‐PDLSCs. (B) Heatmap of the top 10 downregulated and top 10 upregulated lncRNAs in I‐PDLSCs compared with H‐PDLSCs. Red: upregulated lncRNAs in I‐PDLSCs; Green: downregulated lncRNAs in I‐PDLSCs. (C, D) The expression of selected lncRNAs (*SLC30A4‐AS1* and *LINC01614*) was verified via qRT–PCR. (E, F) The knockdown efficiency of *SLC30A4‐AS1* (E) and *LINC01614* (F) in PDLSCs was measured by qRT–PCR. (G, H) After knocking down *SLC30A4‐AS1* (G) or *LINC01614* (H), the expression levels of senescence‐related genes (*P53*, *P27*, *P21*, and *P16*) in PDLSCs were measured via qRT–PCR. The data are presented as the means ± SDs (n = 3). *p < 0.05, **p < 0.01, and ***p < 0.001 represent significant differences between the indicated columns.

Given that lncRNAs serve as critical modulators of cellular senescence [29, 30, 31], we performed a microarray analysis to identify the distinctively altered lncRNAs expression among H‐PDLSCs and I‐PDLSCs. The results showed that 1220 lncRNAs were significantly upregulated and 1564 lncRNAs were significantly downregulated in I‐PDLSCs compared with H‐PDLSCs (Figure 2A). The heatmap shows the top 10 lncRNAs with upregulated expression and the top 10 lncRNAs with downregulated expression (Figure 2B and Table S4). According to the absolute value of fold change, RNA length < 2000 nt, original signal intensity > 100, no overlap with the coding transcript and inclusion in the database (in the GENCODE or RefSeq public databases), six upregulated lncRNAs (*SLC30A4‐AS1*, *AL844908.1*, *LRRN3*, *AL390957.1*, *AC079298.1*, and *AC079298.3*) (Figures 2C and S4A) and six downregulated lncRNAs (*LINC01614*, *AC017076.1*, *LINC01711*, *MIR4432HG*, *LINC02244*, and *APCDD1L‐DT*) (Figures 2D and S4B) were selected, and their expression was validated by qRT–PCR. The results showed that six lncRNAs (*SLC30A4‐AS1*, *AL844908.1*, *LRRN3*, *AC079298.1*, *AC079298.3*, and *LINC01614*) exhibited the same trends as observed in the microarray results with significant differences. Among the upregulated lncRNAs, *SLC30A4‐AS1* exhibited the most significant change in expression, and among the downregulated lncRNAs, only *LINC01614* exhibited a significant change in expression. Therefore, we selected *SLC30A4‐AS1* and *LINC01614* for further study.

Considering that *SLC30A4‐AS1* expression was significantly increased under inflammatory conditions (Figure 2C), *SLC30A4‐AS1* expression was silenced using small interfering RNA (siRNA) and antisense oligonucleotide (ASO) in cells that were cultured under inflammatory conditions. In contrast, *LINC01614* expression was significantly reduced under inflammatory conditions (Figure 2D), so *LINC01614* expression was silenced using siRNA and ASO under non‐inflammatory conditions. After verifying the knockdown of each lncRNA in PDLSCs (Figure 2E,F), the expression of senescence‐related genes in siRNA‐transfected PDLSCs was evaluated by qRT–PCR. Inhibition of *SLC30A4‐AS1* expression significantly affected the expression levels of senescence‐related genes (*P53*, *P21*, and *P16*) under inflammatory conditions (Figure 2G), whereas inhibition of *LINC01614* expression affected only the expression level of *P21* in PDLSCs under non‐inflammatory conditions (Figure 2H). We concluded that *SLC30A4‐AS1* is closely related to cellular senescence. *SLC30A4‐AS1* is *SLC30A4* antisense RNA 1. Studies have found that *SLC30A4* is related to inflammation and brain correlation [32, 33, 34], but there are no relevant reports on the functional study of *SLC30A4*‐*AS1*. Therefore, this study aimed to investigate the biological function of *SLC30A4‐AS1* in PDLSC senescence as well as the potential molecular mechanism.

### 3.4 Knockdown and Overexpression of SLC30A4‐AS1 Can Regulate PDLSC Senescence

**FIGURE 3:**
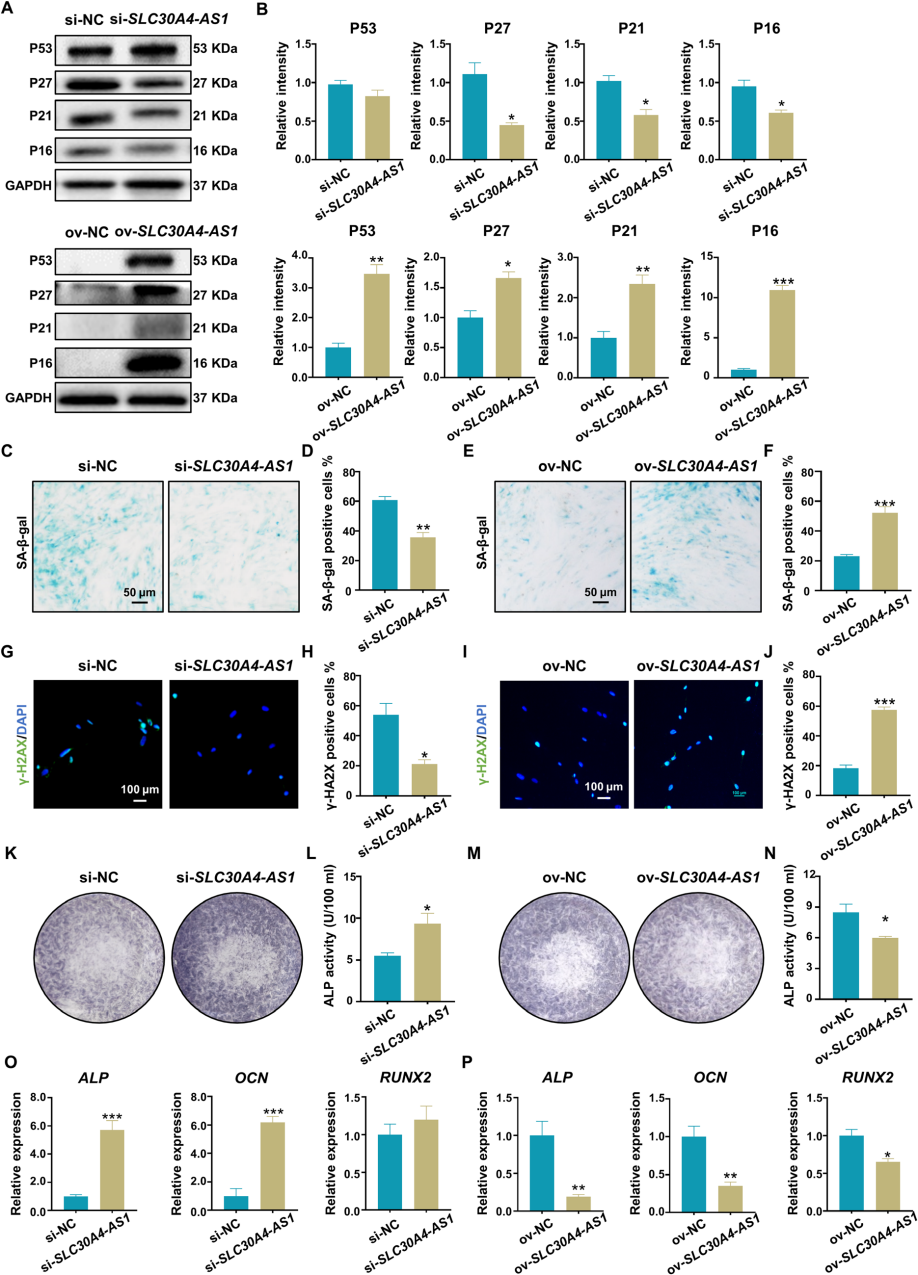
Downregulation/overexpression of *SLC30A4‐AS1* influenced PDLSC senescence under inflammatory conditions. (A) The expression of senescence‐related proteins (P53, P27, P21, and P16) in *SLC30A4‐AS1*‐knockdown (top) or *SLC30A4‐AS1*‐overexpressing (bottom) cells. (B) Semiquantitative analysis of protein expression levels normalised to GAPDH expression levels. (C, E) Senescence‐associated β‐galactosidase was analysed via SA‐β‐gal staining in *SLC30A4‐AS1*‐knockdown (C) or *SLC30A4‐AS1*‐overexpressing (E) cells (scale bar: 50 μm). (D, F) Quantification of SA‐β‐Gal staining positivity in *SLC30A4‐AS1*‐knockdown (D) or *SLC30A4‐AS1*‐overexpressing (F) cells. (G, I) Immunofluorescence analysis of γ‐H2AX expression in *SLC30A4‐AS1*‐knockdown (G) or *SLC30A4‐AS1*‐overexpressing (I) cells. Nuclei were labelled with DAPI (scale bar: 100 μm). (H, J) Percentage of γ‐H2AX‐positive cells when *SLC30A4‐AS1* was knocked down (H) or overexpressed (J). (K, M) Representative image of ALP staining of PDLSCs transfected with si‐*SLC30A4‐AS1* (K) or ov‐*SLC30A4‐AS1* (M) after 14 days of osteogenic differentiation induction. (L, N) Quantification of ALP activity in PDLSCs transfected with si‐*SLC30A4‐AS1* (L) or ov‐*SLC30A4‐AS1* (N) after osteogenic differentiation induction for 14 days. (O, P) Expression levels of osteogenesis‐related genes (*ALP*, *OCN*, and *RUNX2*) in *SLC30A4‐AS1*‐knockdown (O) or *SLC30A4‐AS1*‐overexpressing (P) cells after osteogenic differentiation induction were measured via qRT–PCR. The data are presented as the means ± SDs (*n* = 3). *p < 0.05, **p < 0.01, and ***p < 0.001 represent significant differences between the indicated columns.

To determine whether *SLC30A4‐AS1* could regulate the senescence of PDLSCs, we knocked down *SLC30A4‐AS1* in cells that were cultured under inflammatory conditions (Figure 2E) and overexpressed *SLC30A4‐AS1* in cells that were cultured under non‐inflammatory conditions (Figure S5A) to observe the changes in PDLSC senescence. The proliferation of si‐*SLC30A4‐AS1*‐transfected cells was significantly faster than that of si‐NC‐transfected cells. However, the proliferation of ov‐*SLC30A4‐AS1*‐transfected cells was significantly slower than that of si‐NC‐transfected cells (Figure S5B), but there was no significant difference in PDLSC apoptosis regardless of whether *SLC30A4‐AS1* was knocked down or overexpressed (Figure S5C). Knockdown of *SLC30A4‐AS1* reduced the protein expression levels of P27, P21, and P16 in PDLSCs under inflammatory conditions (Figure 3A,B). However, compared with those in the ov‐NC transfection group, the expression levels of the *P27* and *P21* genes in the ov‐*SLC30A4‐AS1* transfection group were significantly increased under non‐inflammatory conditions (Figure S5D), and the protein expression levels of P53, P27, P21 and P16 in PDLSCs were significantly increased (Figure 3A,B). Inhibition of *SLC30A4‐AS1* reduced β‐galactosidase activity under inflammatory conditions (Figure 3C,D), while β‐galactosidase activity was increased in *SLC30A4‐AS1*‐overexpressing PDLSCs under non‐inflammatory conditions (Figure 3E,F). Immunofluorescence staining revealed that the expression of γ‐H2AX was decreased after *SLC30A4‐AS1* was knocked down (Figure 3G,H) but increased after *SLC30A4‐AS1* was overexpressed (Figure 3I,J). Next, we induced PDLSCs to undergo osteogenic differentiation for 14 days. Knockdown of *SLC30A4‐AS1* expression significantly promoted the osteogenic differentiation of PDLSCs. ALP staining and quantification revealed that, compared with that in the si‐NC group, the ALP activity in the *SLC30A4‐AS1*‐silenced group was significantly increased (Figure 3K,L). However, ALP activity was significantly reduced after the overexpression of *SLC30A4‐AS1* (Figure 3M,N). In addition, after *SLC30A4‐AS1* was knocked down, the expression levels of osteogenic differentiation‐related genes, such as *ALP* and *OCN*, were significantly enhanced (Figure 3O), and when *SLC30A4‐AS1* was upregulated, the gene expression levels of *ALP*, *OCN*, and *RUNX2* were significantly decreased (Figure 3P). Taken together, these data suggest that knockdown or overexpression of *SLC30A4‐AS1* can regulate PDLSC senescence.

### 3.5 SLC30A4‐AS1 Affects the Alternative Splicing of TP53BP1

**FIGURE 4:**
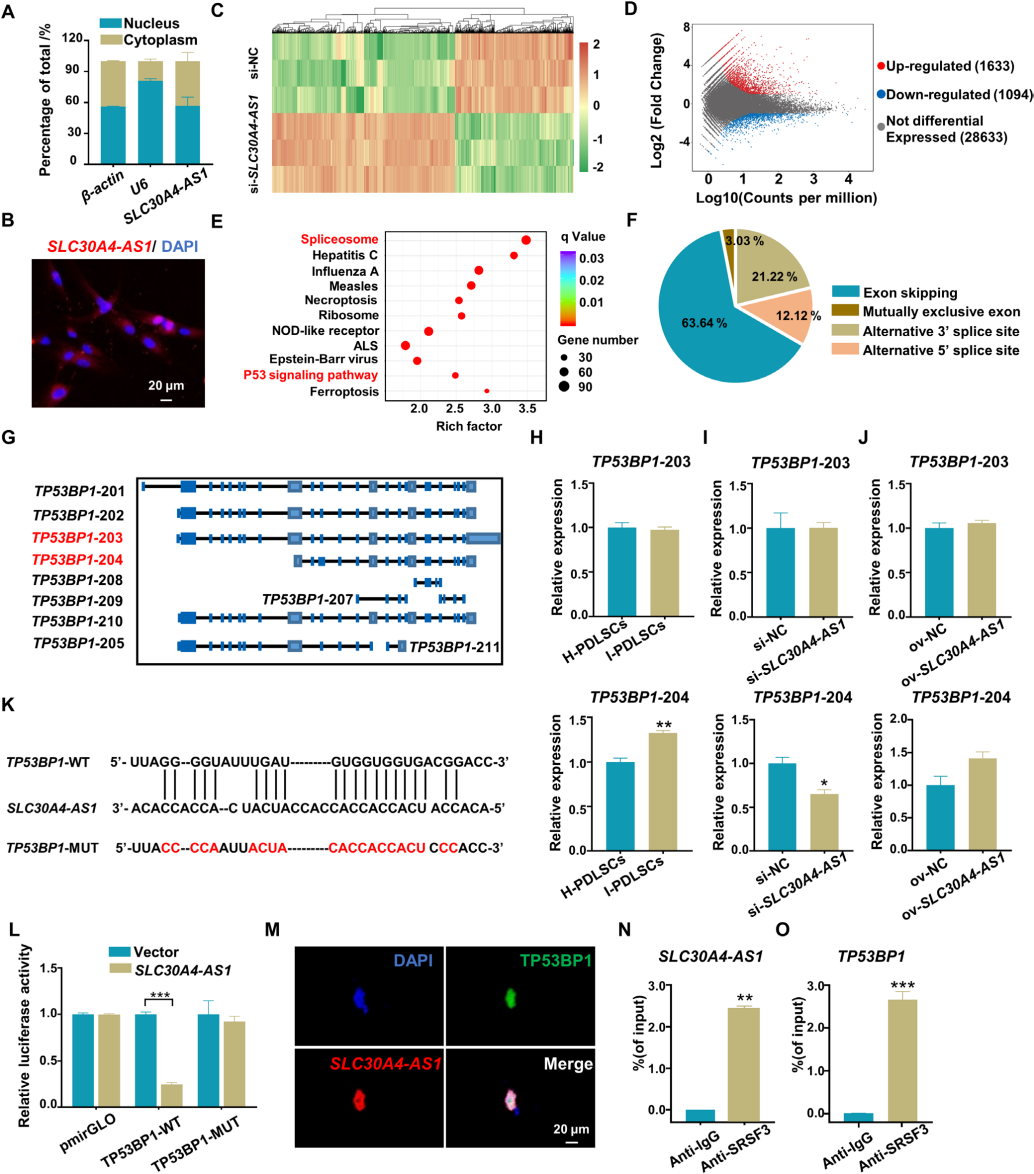
*SLC30A4‐AS1* induces changes in the transcription and AS of *TP53BP1*. (A) Proportions of *SLC30A4‐AS1* transcripts in the nuclear and cytoplasmic fractions of PDLSCs were measured by qRT–PCR; *U6* and *β‐actin* served as the nuclear and cytoplasmic controls, respectively. (B) Subcellular localization of *SLC30A4‐AS1* (red) was determined by FISH, nuclei were stained with DAPI (scale bar: 20 μm). (C) Cluster diagram of the expression patterns of differentially expressed transcripts in PDLSCs after knocking down *SLC30A4‐AS1*. (D) Volcano plots of differentially expressed transcripts in PDLSCs with *SLC30A4‐AS1*‐knockdown. (E) KEGG pathway analysis of the differentially expressed transcripts in PDLSCs with *SLC30A4‐AS1‐*knockdown. (F) Quantification of the different AS events regulated by *SLC30A4‐AS1*. (G) Annotation tracks of *TP53BP1* mRNA transcripts detected in PDLSCs. Boxes and lines represent exons and introns, respectively. Some introns are not drawn to scale. (H) Expression levels of *TP53BP1‐*203 and *TP53BP1‐*204 in H‐PDLSCs and I‐PDLSCs. (I) Expression levels of *TP53BP1‐*203 and *TP53BP1‐*204 in *SLC30A4‐AS1*‐knockdown PDLSCs. (J) Expression levels of *TP53BP1‐*203 and *TP53BP1‐*204 in *SLC30A4‐AS1*‐overexpressing PDLSCs. (K) The predicted binding sites of *TP53BP1* and *SLC30A4‐AS1* as determined by StarBase V3.0. Red indicates binding site mutations. (L) Dual luciferase assays with HEK‐293 T cells transfected with pmirGLO carrying the wild‐type or mutant constructs and the *SLC30A4‐AS1* mimic. (M) Representative fluorescence images showing the co‐localization of *SLC30A4‐AS1* (red) and TP53BP1 (green), nuclei were stained with DAPI (scale bar: 20 μm). (N) The enrichment of *SLC30A4‐AS1* immunoprecipitated by anti‐SRSF3 antibodies was determined by RIP assays (IgG antibodies were used as the control). (O) The enrichment of *TP53BP1* immunoprecipitated by anti‐SRSF3 antibodies was determined by RIP assays (IgG antibodies were used as the control). The data are presented as the means ± SDs (*n* = 3). *p < 0.05, **p < 0.01, and ***p < 0.001 represent significant differences between the indicated columns.

The significance of lncRNAs' functionality is intricately linked with their subcellular localizations [35]. Consequently, we executed subcellular fractionation experiments to evaluate the distribution of *SLC30A4‐AS1*. RNA that was extracted from the nuclear and cytoplasmic fractions was identified using nuclear (*U6*) and cytoplasmic (*β‐actin*) markers. The results showed that *SLC30A4‐AS1* was localised to the cytoplasm and nucleus of PDLSCs (Figure 4A). Similar results were observed in cells that were analysed using fluorescence in situ hybridization (FISH) in situ RNA detection technology (Figure 4B). These observations suggest that *SLC30A4‐AS1* possibly modulates the transcription of target genes [36]. To better understand how *SLC30A4‐AS1* affects PDLSC senescence, we performed full‐length transcriptome sequencing after knockdown of *SLC30A4‐AS1* in PDLSCs, and we analysed the AS events that were regulated by *SLC30A4‐AS1*. After silencing *SLC30A4‐AS1*, the gene transcription patterns were significantly changed (Figure 4C). Among the differentially expressed transcripts, 1683 transcripts were upregulated, and 1094 transcripts were downregulated (Figure 4D). The differentially expressed transcripts were involved in mainly KEGG pathways such as the spliceosome and P53 pathways (Figure 4E). Next, we identified 33 AS events (ΔPSI > 0.45, *p* value < 0.05) (Table S5 and Figure 4F). Among the most prominent AS events, *TP53BP1* (Gene ID ENSG00000067369) was noted. TP53BP1 is a P53‐binding protein 1 that interacts with P53 [37]. Queries in Ensemble revealed that 17 transcripts of *TP53BP1* were present, and 10 of these transcripts encoded proteins. Sequencing revealed 10 transcripts that were present in PDLSCs (Figure 4G). *TP53BP1‐*203 is the default transcript of the human *TP53BP1* gene. Interestingly, the extent of splicing in *TP53BP1*‐204, which includes an early stop codon that results in truncation of the protein at the C‐terminus, was significantly altered after *SLC30A4‐AS1* was knocked down. This mRNA fragment is typically deactivated by cellular mechanisms involving nonsense‐associated RNA decay [38]. However, the level of this truncated protein may increase in certain cells that acquire senescent or other abnormal phenotypes. For example, the RNA and protein levels of TP53β, which is an isoform of TP53, are increased in senescent cells [15, 39]. Similar phenomena may occur with *TP53BP1* transcripts. *TP53BP1*‐204 has a unique sequence between 560 and 709 bp site, while *TP53BP1*‐203 does not have on repetitive coverage fragments with other transcripts after 6232 bp site. We designed specific primers for transcripts and found that compared with those in H‐PDLSCs, the expression of *TP53BP1*‐203 was unchanged, but the expression of *TP53BP1*‐204 was significantly increased in I‐PDLSCs (Figure 4H). Knockdown of *SLC30A4‐AS1* significantly reduced the expression of *TP53BP1*‐204 (Figure 4I), but overexpression of *SLC30A4‐AS1* increased the expression of *TP53BP1*‐204 (Figure 4J). Neither the overexpression nor the knockdown of *SLC30A4‐AS1* affected the expression of *TP53BP1*‐203. Therefore, the increased expression of *TP53BP1*‐204 may be related to the senescence of PDLSCs.

To determine whether *SLC30A4‐AS1* can affect the AS of *TP53BP1*, we performed a luciferase reporter assay in HEK‐293 T cells and used StarBase V3.0 to predict that *SLC30A4‐AS1* interacts with *TP53BP1* (Figure 4K). Overexpression of *SLC30A4‐AS1* inhibited luciferase activity in cells harbouring the *TP53BP1* wild‐type (WT) construct but not in cell harbouring the mutant (MUT) construct (Figure 4L). We also investigated *SLC30A4‐AS1* co‐localised with TP53BP1 by FISH and immunofluorescence imaging. *SLC30A4‐AS1* exhibited a punctate pattern and co‐localised with TP53BP1 in the nuclei of PDLSCs (Figure 4M). These findings indicated that *SLC30A4‐AS1* bound to *TP53BP1*. *SLC30A4‐AS1* does not have the ability to splice by itself, and it needs to bind to splicing factors to regulate mRNA splicing. Serine/arginine splicing factors (SRSFs) are important components of the spliceosome and are essential for AS [40]. We found significant transcriptional differences in SRSF3 in the full‐length transcriptome sequencing (Table S5), which suggested that SRSF3 was actively involved in the process of *TP53BP1* alternative splicing regulated by *SLC30A4‐AS1*. SRSF3 is essential for AS during senescence has been reported [41, 42, 43]. To investigate the specificity of the interaction among *SLC30A4‐AS1*, *TP53BP1*, and SRSFs, an RNA immunoprecipitation (RIP) assay was performed on nuclear extracts using an anti‐SRSF3 antibody. qRT‐PCR analysis was performed on the RIP samples that were precipitated by anti‐SRSF3 or anti‐IgG antibodies using *SLC30A4‐AS1*‐specific primers, and the results showed that *SLC30A4‐AS1* specifically interacts with SRSF3 in PDLSCs (Figure 4N). Similarly, *TP53BP1* was found to interact specifically with SRSF3 using *TP53BP1*‐specific primers (Figure 4O). These experiments demonstrated that *SLC30A4‐AS1* regulates *TP53BP1* mRNA splicing through SRSF3, and overexpression or knockdown of *SLC30A4‐AS1* affected the expression of *TP53BP1*‐204. So there is an upstream and downstream relationship between *SLC30A4‐AS1* and *TP53BP1*.

### 3.6 The Expression of the TP53BP1‐204 Transcript Affects PDLSC Senescence

**FIGURE 5:**
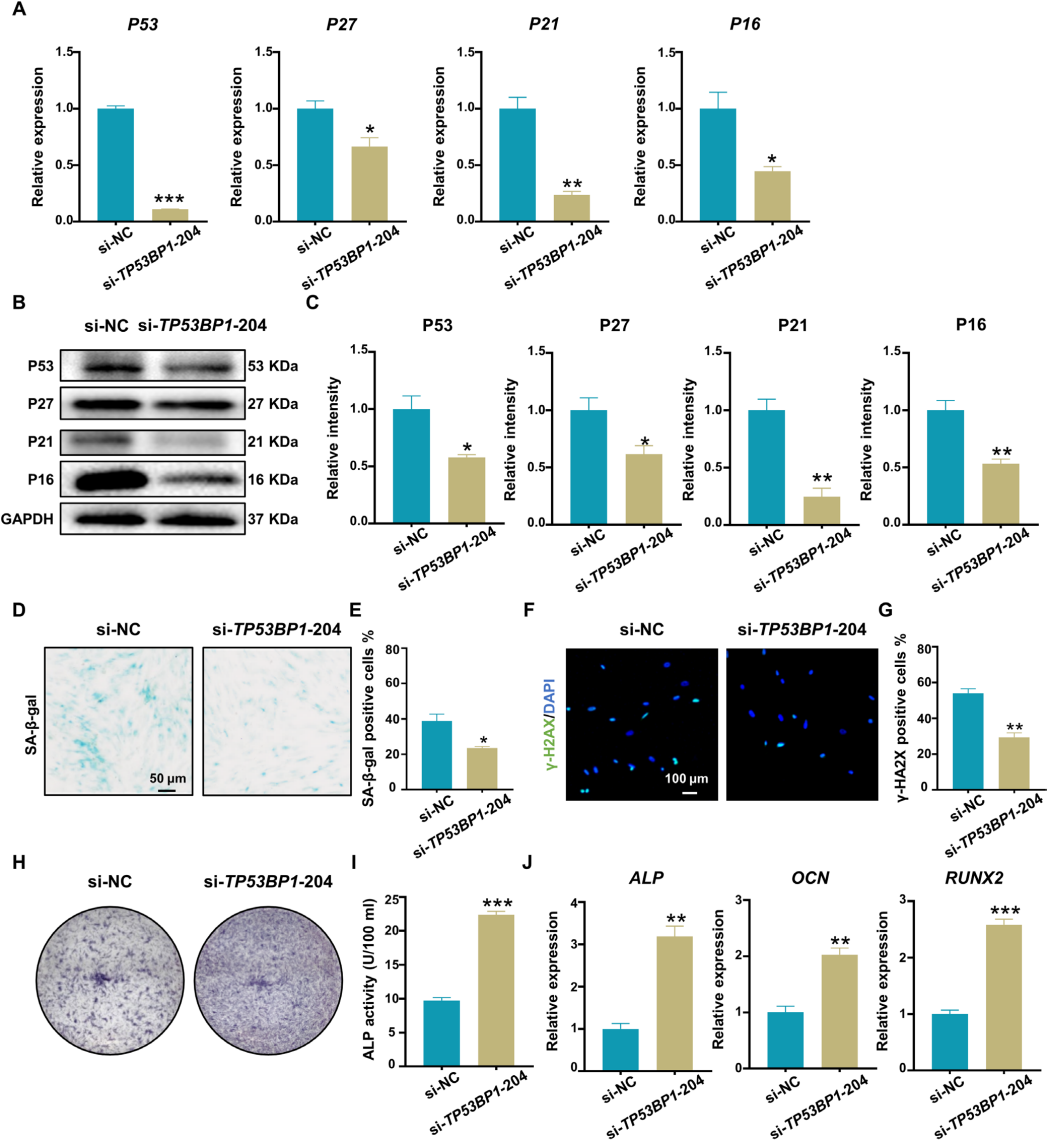
Downregulation of *TP53BP1‐*204 ameliorated PDLSC senescence under inflammatory conditions. (A) Expression levels of senescence‐related genes (*P53*, *P27*, *P21*, and *P16*) in PDLSCs transfected with si‐*TP53BP1*‐204 were measured via qRT–PCR. (B) The expression of senescence‐related proteins (P53, P27, P21, and P16) in PDLSCs after *TP53BP1‐*204 was knocked down. (C) Semiquantitative analysis of protein expression levels normalised to GAPDH expression levels. (D) Senescence‐associated β‐galactosidase in *TP53BP1‐*204‐knockdown PDLSCs was analysed via SA‐β‐Gal staining (scale bar: 50 μm). (E) Quantification of SA‐β‐Gal staining in *TP53BP1‐*204‐knockdown PDLSCs. (F) Immunofluorescence staining for γ‐H2AX in *TP53BP1‐*204‐knockdown PDLSCs. Nuclei were labelled with DAPI (scale bar: 100 μm). (G) Percentage of γ‐H2AX‐positive *TP53BP1‐*204‐knockdown PDLSCs. (H) Representative image of ALP staining in PDLSCs transfected with si‐*TP53BP1‐*204 after 14 days of osteogenic differentiation induction. (I) Quantification of ALP activity in PDLSCs transfected with si‐*SLC30A4‐AS1* after osteogenic induction for 14 days. (J) Expression levels of osteogenesis‐related genes (*ALP*, *OCN* and *RUNX2*) in *TP53BP1‐*204‐knockdown PDLSCs after osteogenic induction were measured via qRT–PCR. The data are presented as the means ± SDs (*n* = 3). *p < 0.05, **p < 0.01, and ***p < 0.001 represent significant differences between the indicated columns.

To verify whether the *TP53BP1* transcripts are associated with PDLSC senescence, we used specific siRNAs to knock down *TP53BP1*‐204 but not *TP53BP1*‐203 (Figure S6A). After *TP53BP1*‐204 knockdown, the expression of the genes *P53*, *P27*, *P21*, and *P16* was significantly decreased in PDLSCs that were cultured under inflammatory conditions (Figure 5A). Similarly, the levels of senescence‐related proteins were significantly reduced (Figure 5B,C). Accordingly, SA‐β‐gal activity was significantly reduced after *TP53BP1*‐204 knockdown (Figure 5D,E). Immunofluorescence analysis revealed that inhibition of *TP53BP1*‐204 decreased the expression of γ‐H2AX under inflammatory conditions (Figure 5F,G). After the induction of osteogenic differentiation, ALP staining and quantification showed that the ALP activity in PDLSCs increased after *TP53BP1*‐204 inhibition (Figure 5H,I). Similarly, qRT–PCR analysis revealed increased levels of the osteogenesis‐related genes *ALP*, *OCN*, and *RUNX2* after knocking down *TP53BP1*‐204 (Figure 5J). These results suggest that the expression of *TP53BP1*‐204 regulates the senescence of PDLSCs.

### 3.7 Inhibition of TP53BP1‐204 Reverses SLC30A4‐AS1 Overexpression‐Induced PDLSC Senescence

**FIGURE 6:**
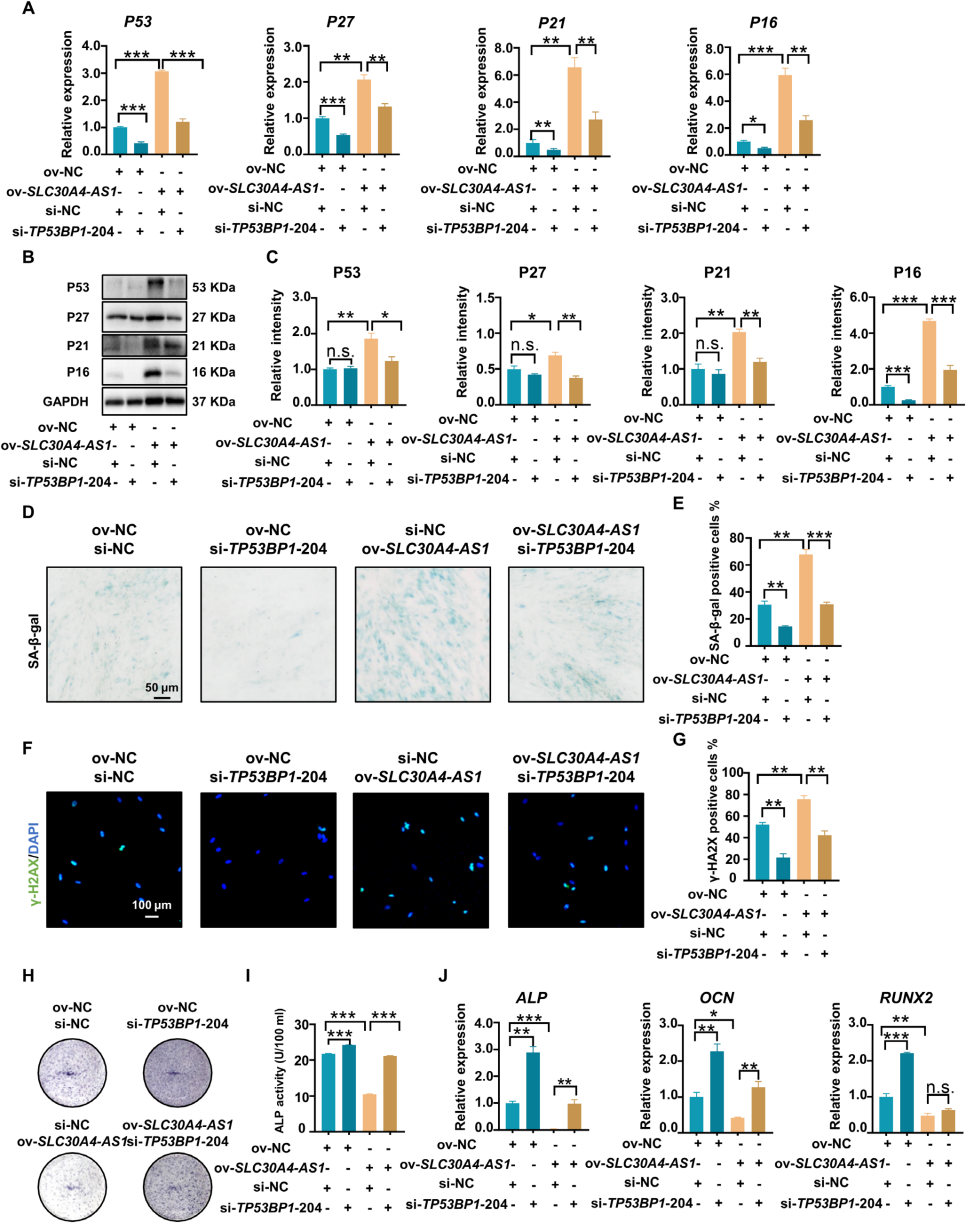
Downregulation of *TP53BP1‐*204 reversed the senescence of *SLC30A4‐AS1*‐overexpressing PDLSCs. (A) Expression levels of senescence‐related genes (*P53*, *P27*, *P21*, and *P16*) in PDLSCs were measured via qRT–PCR. (B) The expression of senescence‐related proteins (P53, P27, P21, and P16) in PDLSCs was determined via western blotting. (C) Semiquantitative analysis of protein expression levels normalised to GAPDH expression levels. (D) Senescence‐associated β‐galactosidase in PDLSCs was analysed via SA‐β‐gal staining (scale bar: 50 μm). (E) Quantification of SA‐β‐Gal staining in PDLSCs. (F) Immunofluorescence staining for γ‐H2AX in PDLSCs; nuclei were stained with DAPI (scale bar: 100 μm). (G) Percentage of γ‐H2AX‐positive PDLSCs. (H) Representative image of ALP staining of PDLSCs after 14 days of osteogenic induction (scale bar: 50 μm). (I) Quantification of ALP activity in PDLSCs after osteogenic induction for 14 days. (J) The expression levels of osteogenesis‐related genes (*ALP*, *OCN* and *RUNX2*) in PDLSCs after osteogenic induction were measured via qRT–PCR. The data are presented as the means ± SDs (*n* = 3). *p < 0.05, **p < 0.01, and ***p < 0.001 represent significant differences between the indicated columns.

To determine whether *SLC30A4‐AS1*‐mediated cellular senescence was associated with the expression of *TP53BP1* transcripts, *TP53BP1*‐204 was silenced using siRNA in *SLC30A4‐AS1*‐overexpressing cells (Figure S7A). Functional studies showed that knockdown of *TP53BP1*‐204 ameliorated the *SLC30A4‐AS1* overexpression‐induced increase in senescence‐related protein and gene expression (Figure 6A–C). Similarly, inhibition of *TP53BP1*‐204 abolished the *SLC30A4‐AS1* overexpression‐induced increase in β‐galactosidase activity and γ‐H2AX expression in PDLSCs, as shown by SA‐β‐gal staining and immunofluorescence analysis (Figure 6D–G). Inhibition of *TP53BP1*‐204 reversed the *SLC30A4‐AS1* overexpression‐induced decrease in ALP activity, as shown by ALP staining and quantification (Figure 6H,I). Consistent with these findings, qRT–PCR analysis revealed that *TP53BP1*‐204 knockdown improved the effect of *SLC30A4‐AS1* overexpression on reducing *ALP*, *OCN*, and *RUNX2* gene expression (Figure 6J). Therefore, the role of *SLC30A4‐AS1* in regulating PDLSC senescence is related to the expression of *TP53BP1*‐204.

### 3.8 SLC30A4‐AS1 and TP53BP1‐204 Act in Concert to Regulate PDLSC Senescence and Osteogenic Differentiation In Vivo

**FIGURE 7:**
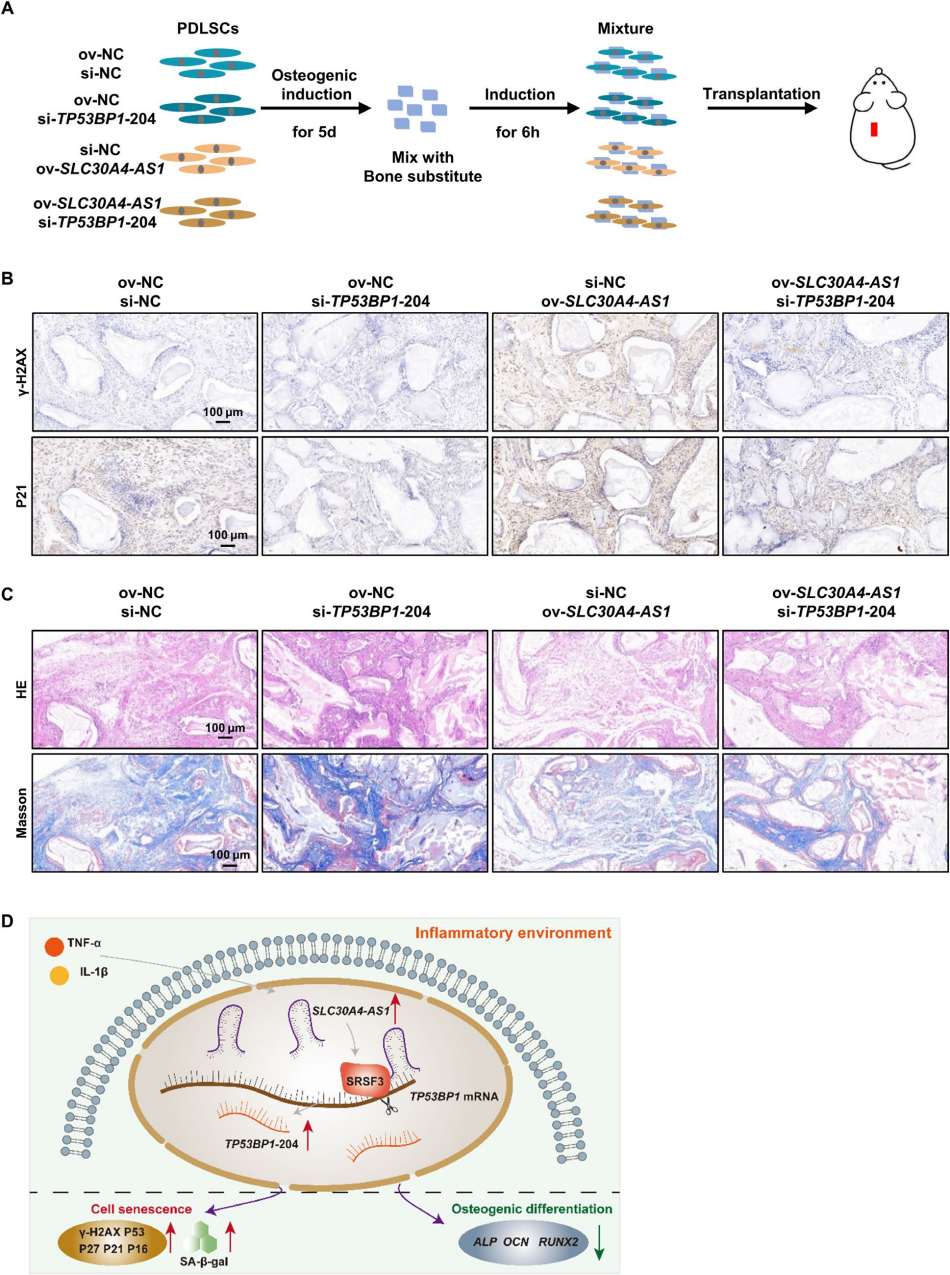
*SLC30A4‐AS1* and *TP53BP1‐*204 regulated PDLSC senescence in vivo. (A) Schematic diagram of the establishment of the subcutaneous xenograft model in BALB/c nude mice. (B) Immunohistochemistry staining for γ‐H2AX (top) and P21 (bottom) in transplants derived from the PDLSC‐Bio‐OSS mixtures (scale bar: 100 μm). (C) HE (top) and Masson (bottom) staining of transplants derived from the PDLSC‐Bio‐OSS mixtures (scale bar: 100 μm). (D) The diagram shows that the *SLC30A4‐AS1* mediates the AS of *TP53BP1* to regulate PDLSC senescence under inflammatory conditions.

We also observed the effects of *SLC30A4‐AS1* and *TP53BP1*‐204 on the senescence and osteogenic differentiation of PDLSCs in nude mice in vivo (Figure 7A). Immunohistochemistry revealed that, compared with those in the control grafts, the expression levels of P21 and γ‐H2AX were decreased in the *TP53BP1*‐204‐knockdown grafts. However, the expression levels of P21 and γ‐H2AX were increased in the *SLC30A4‐AS1*‐overexpressing grafts, and these increases were ameliorated when *TP53BP1*‐204 was knocked down (Figure 7B). The results of HE staining and Masson staining showed that PDLSCs that were subcutaneously transplanted into nude mice formed mainly collagen fibres, and there were scattered gaps between the tissues that were formed by bone powder dissolved after decalcification. Compared with those in the control group, the collagen fibres in the *TP53BP1*‐204‐knockdown group were denser, and mineralized matrix deposition was observed between some collagen fibres, which was consistent with the characteristics of early bone formation; however, the collagen fibres in the *SLC30A4‐AS1‐*overexpressing group were sparse and lacked matrix deposition between collagen fibres (Figure 7C). Knockdown of *TP53BP1*‐204 improved the ability of PDLSCs to form periodontal ligament/bone‐like tissues in nude mice, and this effect was weakened by *SLC30A4‐AS1* overexpression. These results indicated that *SLC30A4‐AS1* regulated the expression of *TP53BP1* transcripts under inflammatory conditions, thus affecting the senescence of PDLSCs and subsequently affecting the regenerative potential of PDLSCs (Figure 7D).

## 4 Discussion

The periodontal ligament stands as an exceptional form of specialised connective tissue tasked with affixing the dentition securely onto the alveolar bone, thereby conferring teeth with essential defence against the stressors imparted by the masticatory apparatus [44]. PDLSCs play important roles as progenitor cells in the process of periodontal regenerations [45]. P‐PDLSCs are isolated from the periodontal ligament tissues of patients with periodontitis [9], but due to the influence of the inflammatory microenvironment in vivo, some biological properties of P‐PDLSCs are significantly disrupted, which in turn affects their tissue regeneration ability [8, 10]. The impaired immune regulation and differentiation of P‐PDLSCs largely limit the direct application of these cells in cell therapy and tissue engineering [6, 46]. In this study, we aimed to describe inflammation‐induced PDLSC senescence, elucidate its effect on tissue regeneration, and identify the key lncRNAs that are involved in PDLSC senescence under inflammatory conditions. Moreover, how these molecules mediate the aberrant AS of aging‐related proteins were investigated, and new targets for treating inflammation‐induced cellular dysfunction were identified.

Previous research has indicated that PDLSCs derived from periodontal disease exhibit diminished osteogenic capabilities [47]. In addition, studies have shown that inflammation can promote cell senescence [48] and that cell senescence can reduce cell function. Therefore, we hypothesized that the inflammatory environment accelerates PDLSC senescence and leads to a reduction in their regenerative potential. In this study, we harvested P‐PDLSCs and H‐PDLSCs from the same periodontitis patient and confirmed that P‐PDLSCs exhibited senescence and decreased regeneration ability compared with H‐PDLSCs (Figure S2). TNF‐α and IL‐1β are pro‐inflammatory cytokines that are involved in the initiation and progression of periodontitis and have been used to establish models of inflammatory environments [49, 50]. Previous studies have shown that stimulating cells with inflammatory factors accelerates cell proliferation [51, 52]. However, these experiments mainly involved short‐term inflammatory factor stimulation, which may lead to temporary stress‐induced cell proliferation.

LncRNAs intricately interface within multiple biological facets, premierly influencing cellular regulatory mechanisms encompassing proliferation, differentiation, apoptosis, and senescence [53]. However, lncRNAs that are involved in the regulation of inflammation‐induced PDLSC senescence remain to be discovered and studied. In this study, we used microarray analysis to explore the differential expression of lncRNAs under inflammatory and control conditions. However, not all differentially expressed lncRNAs can play roles in complex regulatory mechanisms. After careful screening, we identified *SLC30A4‐AS1* among the 12 validated lncRNAs. *SLC30A4‐AS1* exhibited the most significant change in expression, and its function has not been previously explored. Further gain/loss‐of‐function studies showed that *SLC30A4‐AS1* is a regulator that is associated with inflammation‐mediated PDLSC senescence (Figure 3). At the molecular level, lncRNAs can regulate mRNA transcription by altering mRNA splicing [20]. Most human genes undergo AS in a variety of physiological and pathological processes [54], and studies have shown that the dysregulated splicing of many precursor mRNAs is associated with senescence [55, 56, 57]. In this study, we screened AS events that are mediated by *SLC30A4‐AS1* in PDLSCs.

Among the genes with different transcription patterns, we focused on *TP53BP1*. *TP53BP1* plays an important role in DNA damage repair, it is also closely related to cell senescence [58]. To date, the expression of TP53BP1 transcripts during cellular senescence and the molecular mechanisms and physiological relevance of their regulation have not been fully elucidated. The default transcript of the human TP53BP1 gene is *TP53BP1*‐203. However, *TP53BP1*‐204 expression was significantly increased in senescent PDLSCs (Figure 4). After knockdown/overexpression of *SLC30A4‐AS1*, the expression of *TP53BP1*‐204 was altered, but the expression of *TP53BP1*‐203 did not change. Furthermore, the binding of *SLC30A4‐AS1* to the *TP53BP1* precursor mRNA and splicing protein SRSF3 was confirmed by FISH, dual luciferase reporter gene assay, and RIP (Figure 4), and these results confirmed that *SLC30A4‐AS1* regulated the AS of *TP53BP1*. We found that the knockdown of *TP53BP1*‐204 under inflammatory conditions increased the expression levels of senescence‐related proteins and genes in PDLSCs (Figure 5), which indicated that the accumulation of *TP53BP1*‐204 was related to cell senescence. Furthermore, knockdown of *TP53BP1*‐204 ameliorated *SLC30A4‐AS1* overexpression‐induced PDLSC senescence (Figure 6), and similar effects were demonstrated in vivo (Figure 7). This indicates that *SLC30A4‐AS1* and *TP53BP1*‐204 play key regulatory roles in PDLSC senescence under inflammatory conditions. Unfortunately, there are no commercially available antibodies against *TP53BP1*‐204 and *TP53BP1*‐203, so this study only validated and examined the functions of *TP53BP1*‐204 and *TP53BP1*‐203 at the RNA level. Further studies are needed to determine the expression and function of *TP53BP1*‐204 and *TP53BP1*‐203 encoded proteins and the function and regulation of other TP53BP1 transcripts.

Taken together, the results of our study demonstrated for the first time that *SLC30A4‐AS1* regulates the AS of *TP53BP1*, resulting in increased expression of the *TP53BP1*‐204 transcript under inflammatory conditions. *SLC30A4‐AS1*‐mediated *TP53BP1* AS plays an important role in the regulation of PDLSC senescence. It has been previously reported that TP53BP1 affects cellular senescence by repairing DNA damage and triggering the P53 response. Our findings provide new insights into how *TP53BP1* regulates cellular senescence as well as cellular and molecular events during inflammation‐mediated cellular senescence and identify a novel target for improving the regenerative potential of diseased PDLSCs.

## Author Contributions

M.X., D.G., and X.‐Y.Z.: experimental design and execution, financial support, data acquisition and analysis, and manuscript writing. X.‐T.H., R.X.W., Y.Y., and F.‐M.C: experimental execution, financial support, data analysis and technical assistance. R.J., L.L., and Y.‐J.T.: cell culture and animal experiment. X.L. and B.‐M.T.: experimental conception and design, financial support, data analysis, and final approval of the manuscript.

## Ethics Statement

All experimental protocols dealing with human subjects were approved by the Ethics Committee of the Stomatological Hospital of FMMU (Title of the project: Effect of inflammatory microenvironment on the differentiation of periodontal ligament stem cells and its mechanism. Approval number: IRB‐REV‐2022120. Date of approval: 8/10/2022). The animal research protocol was approved by the Laboratory Animal Welfare & Ethical Committee, School of Stomatology, Air Force Military Medical University. (Title of the project: *SLC30A4‐AS1* regulates *TP53BP1* alternative splice variant expression to mediate periodontal ligament stem cell senescence in an inflammatory environment. Approval number: 2023‐kq‐056. Date of approval: 26/10/2023).

## Consent

Informed consent was signed by all the subjects who donated their extracted teeth for cell isolation.

## Conflicts of Interest

The authors declare no conflicts of interest.

## Supporting information


**DATA S1:** Supporting Information.

## Data Availability

The lncRNA microarray data is provided in the GEO databases (accession number GSE260558, https://www.ncbi.nlm.nih.gov/geo/query/acc.cgi?acc=GSE260558, the following secure token has been created to allow review of record GSE260558 while it remains in private status: kfaliwewrlghhgx), and the full‐length transcriptome sequencing data is provided in the SRA databases (accession number PRJNA1082874, https://dataview.ncbi.nlm.nih.gov/object/PRJNA1082874?reviewer=19omeden7253njje3src4e2g8g).
