# Supplementary material for: SLC30A4‐AS1 Mediates the Senescence of Periodontal Ligament Stem Cells in Inflammatory Environments via the Alternative Splicing of TP53BP1
Source: Cell Prolif. 2024 Nov 21;58(4):e13778. doi: 10.1111/cpr.13778 (PMC11969240; doi:10.1111/cpr.13778)
Supplement: Supplementary file 1 — DATA S1: Supporting Information. [file CPR-58-e13778-s001.docx]

Supplementary Data:

**Title**

***SLC30A4-AS1* mediates the senescence of periodontal ligament stem cells in inflammatory environments via the alternative splicing of *TP53BP1***

**Authors**

Mei Xu^a,#^, Dian Gan^a,#^, Xi-Yu Zhang^a,#^, Xiao-Tao He^a^, Rui Xin Wu^a^, Yuan Yin^a^, Rui Jin^a^, Lin Li^a^, Yu-Jie Tan^a^, Fa-Ming Chen^a,^, Xuan Li^a,*^, Bei-Min Tian^a,*^

**Table S1.** Sequences of the primer that were used in this study.

| Gene | Forward 5’-3’ | Reverse 5’-3’ |
| --- | --- | --- |
| *P16* | AAGGTCCCTCAGACATCCCC | AACTACGAAAGCGGGGTGG |
| *P21* | CTGTCACTGTCTTGTACCCTTGT | CCCAGCAGAGGAACCACTACTA |
| *TP53* | TCTGACTGTACCACCATCCACTA | TGTTCCGTCCCAGTAGATTACCA |
| *GAPDH* | AGAGCACAAGAGGAAGAGAGAGA | TGGTTGAGCACAGGGTACTTTAT |
| *P27* | GTCAAACGTAAACAGCTCGAATT | GATGTCCATTCCATGAAGTCAGC |
| *ALP* | CTATCCTGGCTCCGTGCTC | TGGAGACACCCATCCCATCT |
| *Runx2* | TCGCCTCACAAACAACCACA | GCTTGCAGCCTTAAATGACTCT |
| *OCN* | CTCACACTCCTCGCCCTATTG | GCTTGGACACAAAGGCTGCAC |
| *AL844908.1* | TCATGGTTTCAAGTGGGCAGA | AGCCACCCTTCGATCCCAAT |
| *LRRN3* | GCACCTATTTATACCGGGCAAGA | TCTCCTCCTCTTCCTCGTCTTTA |
| *AL390957.1* | ACTCTCCTGCCTCAATTCTACT | CCACAGGTAACGATGAGCCA |
| *AC079298.1* | TCGGCAGTTGACTATCCAATGT | GTGGCAACAGCAACCTCAAG |
| *AC079298.3* | TGCTTCGTGAACCCTGGATG | GAAGGCCTGAATGGGCAAAC |
| *SLC30A4-AS1* | ATCCACACACTCGGACCCTA | TGGCAGTTGCTCACTCTCAG |
| *AC017076.1* | AGTTGAGGTGGCCCTGAATG | ACCCACTGGATTTGTTTTTGAGTT |
| *APCDDIL-DT* | AGGCATTGGGAGGAGAAACG | CCTCAGCATGGGAACACCAA |
| *LINC01711* | GGTCTGGAGCCGTTTCTCTC | ATCCATCCTTGACCCTCGGA |
| *LINC01614* | CTCCAAGGCACCAACAAAGC | GAAGACATCCTCAGCCCACC |
| *MIR4432HG* | CGGATTTTTGGCGTTGAGCA | TCATGCAGTTTCAAGCCCCA |
| *LINC02244* | TCTGGAAGGAGTGTCGGTCT | TTCGTGTTCACAGATTGGGAGA |
| *TP53BP1-*203 | CCAAGGGTTGCTCCTACCTG | CGCCAGTCTTCACTCCTGTT |
| *TP53BP1-*204 | ACAATCATGCCGTCTGGAGT | TCCACTTCTGCATCTGGACA |
| *U6* | TGCTTCGGCAGCACATATAC | TCACGAATTTGCGTGTCATC |
| *β-actin* | CTCCATCCTGGCCTCGCTGT | GCTGTCACCTTCACCGTTCC |

**Table S2.** Antibodies that were used in this study.

| Antibodies | Brands | Cat. No. | Dilution |
| --- | --- | --- | --- |
| CD90-FITC | Invitrogen | 11-0909-41 | 5 µl/106 cells |
| CD105-PE | Invitrogen | 12-1057-41 | 5 µl/106 cells |
| CD146-PE | Invitrogen | 12-1469-41 | 5 µl/106 cells |
| CD34-FITC | Invitrogen | 11-0349-41 | 5 µl/106 cells |
| CD45-FITC | Invitrogen | 11-0459-41 | 5 µl/106 cells |
| CD31-FITC | Invitrogen | 11-0319-41 | 5 µl/106 cells |
| P16 | ProteinTech | 10883-1-AP | 1:5000 |
| P27 | ProteinTech | 25614-1-AP | 1:5000 |
| P21 | SAB | 30427-1 | 1:2000 |
| P53 | SAB | HW109-1 | 1:2000 |
| γ-H2AX | SAB | 54667-1 | 1:100 |
| GAPDH | SAB | 21612-1 | 1:5000 |
| SRSF3 | Santa cruz | sc-13510 | 200 µg/m |
| 53BP1 | Santa cruz | sc-515841 | 200 µg/m |
| Goat anti-Rabbit IgG | SAB | L3012-1 | 1:5000 |
| Goat anti-Mouse IgG | SAB | L3032-1 | 1:5000 |

**Table S3.** siRNA and ASO sequences that were used in this study.

| siRNA or ASO | Sequences |
| --- | --- |
| siRNA-SLC30A4-AS1-1 | 5'-GCAGUUAUAGCUCACUAUA-3' |
|  | 5'-UAUAGUGAGCUAUAACUGC-3' |
| siRNA-SLC30A4-AS1-2 | 5'-CACAGAAUAUCGGAAUCUA-3' |
|  | 5'-UAGAUUCCGAUAUUCUGUG-3' |
| siRNA-SLC30A4-AS1-3 | 5'-GCUCGGAGUAGAAUUGAGA-3' |
|  | 5'-UCUCAAUUCUACUCCGAGC-3' |
| ASO-SLC30A4-AS1 | 5'-TGCAGTGAGCTGAAATTCTG-3' |
| siRNA-LINC01614 | 5'-CCCUUAAAGUCAAGUACAA-3' |
| ASO-LINC01614-1 | 5'-ATCTATGGGTGCAGTACCAG-3' |
| ASO-LINC01614-2 | 5'-TCAATGCAGACTTGCTCCCA-3' |
| siRNA-TP53BP1-204 | 5'-GGAGUCUCCUGCUACCUAU-3' |
|  | 5'-AUAGGUAGCAGGAGACUCC-3' |
| siRNA-NC  ASO-NC | 5'-UUCUCCGAACGUGUCACGUTT-3' |
|  | 5'-ACGUGACACGUUCGGAGAATT-3' |

**Table S4.** Information on the top 10 upregulated and top 10 downregulated lncRNAs in I-PDLSCs compared with H-PDLSCs that were identified in the present study.

| **Gene ID** | **Gene**  **Symbol** | **Regulation** | **P-value** | **Fold Change** | **I-PDLSCs Raw Intensity** | **H-PDLSCs Raw Intensity** |
| --- | --- | --- | --- | --- | --- | --- |
| ENSG00000253522 | MIR3142HG | up | 7.9E-06 | 175.96 | 870.65117 | 5 |
| ENSG00000250945 | AC096773.1 | up | 0.00792 | 92.75 | 901.8012 | 5 |
| ENSG00000267607 | AC011511.5 | up | 4.6E-09 | 92.63 | 456.53397 | 5 |
| ENSG00000272825 | AL844908.1 | up | 8.4E-08 | 92.15 | 454.40508 | 5 |
| ENSG00000173114 | LRRN3 | up | 5.9E-05 | 85.94 | 464.53169 | 5.745827 |
| ENSG00000285280 | AL390957.1 | up | 7.2E-06 | 76.81 | 1261.9354 | 17.349393 |
| ENSG00000278981 | AC079298.1 | up | 4.1E-05 | 56.49 | 291.84983 | 5 |
| ENSG00000280241 | AC079298.3 | up | 1.1E-05 | 50.06 | 251.58554 | 5.084113 |
| ENSG00000259354 | SLC30A4-AS1 | up | 3.9E-05 | 40.13 | 353.39029 | 9.684605 |
| ENSG00000253194 | AL137009.1 | up | 0.0007 | 39.06 | 332.9931 | 9.307047 |
| G046000 | G046000 | down | 0.00242 | 102.33 | 137.61298 | 9609.85567 |
| ENSG00000271947 | AC017076.1 | down | 1.8E-07 | 77.87 | 5 | 404.585233 |
| ENSG00000228203 | RNF144A-AS1 | down | 0.00014 | 57.41 | 9.062464 | 501.476763 |
| ENSG00000231290 | APCDD1L-DT | down | 0.00073 | 48.11 | 8.737696 | 378.355983 |
| ENSG00000268941 | LINC01711 | down | 1.1E-06 | 36.10 | 16.382913 | 620.881523 |
| ENSG00000230838 | LINC01614 | down | 5.3E-06 | 34.54 | 5 | 182.084037 |
| AX747189 | AX747189 | down | 1.6E-05 | 27.34 | 5.558483 | 157.166307 |
| ENSG00000228590 | MIR4432HG | down | 1.5E-05 | 26.39 | 16.625876 | 451.435033 |
| ENSG00000259590 | LINC02244 | down | 7.1E-08 | 24.49 | 5.001633 | 128.74185 |
| ENSG00000196979 | AL360004.1 | down | 6.6E-07 | 24.08 | 5 | 123.683658 |

**Table S5.** Information on the differentially expressed transcripts in PDLSCs transfected with ov-*SLC30A4-AS1* compared with PDLSCs transfected with ov-NC that were identified in the present study.

| **Gene ID** | **Gene** | **Event Type** | **Reference Transcript** | Δ**PSI (%)** | **T test**  **p value** |
| --- | --- | --- | --- | --- | --- |
| ENSG00000112081 | SRSF3 | SES | SRSF3-203 | 79.2 | 4.15E-07 |
| ENSG00000152795 | HNRNPDL | TSS\|A3SS | TSS.ONT.9881.9 | 69.5 | 4.63E-07 |
| ENSG00000152795 | HNRNPDL | SES | HNRNPDL-202 | 68.5 | 7.27E-07 |
| ENSG00000143889 | HNRNPLL | SES | HNRNPLL-201 | 55.2 | 5.81E-04 |
| ENSG00000132773 | TOE1 | A3SS | ONT.208.1 | 54.93 | 2.98E-04 |
| ENSG00000131051 | RBM39 | SES | RBM39-206 | 54.01 | 2.67E-06 |
| ENSG00000131051 | RBM39 | A3SS | ONT.8105.20 | 53.69 | 7.89E-06 |
| ENSG00000148187 | MRRF | MES | MRRF-211 | 51.64 | 2.53E-04 |
| ENSG00000153485 | TMEM251 | A5SS | TMEM251-202 | 50.77 | 2.63E-05 |
| ENSG00000124193 | SRSF6 | SES | SRSF6-202 | 50.11 | 4.27E-05 |
| ENSG00000115875 | SRSF7 | SES | SRSF7-204 | 49.31 | 4.04E-06 |
| ENSG00000116679 | IVNS1ABP | SES | ONT.1246.2 | 49.17 | 2.27E-04 |
| ENSG00000101247 | NDUFAF5 | SES | NDUFAF5-201 | 48.2 | 2.81E-04 |
| ENSG00000100908 | EMC9 | A3SS | EMC9-206 | 48.19 | 8.32E-04 |
| ENSG00000067369 | TP53BP1 | SES | TP53BP1-204 | 47.83 | 1.49E-03 |
| ENSG00000164548 | TRA2A | SES | ONT.11900.1 | 46.4 | 7.13E-06 |
| ENSG00000215126 | CBWD6 | SES | CBWD6-202 | 45.71 | 1.54E-03 |
| ENSG00000111011 | RSRC2 | SES | RSRC2-220 | 45.55 | 1.61E-05 |
| ENSG00000124193 | SRSF6 | A5SS | SRSF6-202 | 45.34 | 5.15E-05 |
| ENSG00000075292 | ZNF638 | SES | ONT.6953.16 | -45.41 | 5.48E-05 |
| ENSG00000082213 | C5orf22 | A3SS | C5orf22-201 | -46.91 | 2.86E-04 |
| ENSG00000082213 | C5orf22 | A3SS | C5orf22-201 | -46.91 | 2.86E-04 |
| ENSG00000136536 | 7-Mar | SES | MARCH7-201 | -47.12 | 2.48E-05 |
| ENSG00000036549 | AC118549.1 | A5SS | AC118549.1-202 | -48.28 | 1.28E-04 |
| ENSG00000115042 | FAHD2A | SES | FAHD2A-201 | -50.02 | 1.50E-04 |
| ENSG00000138074 | SLC5A6 | TSS\|A5SS | TSS.SLC5A6-204 | -51.06 | 8.79E-04 |
| ENSG00000131876 | SNRPA1 | SES | SNRPA1-201 | -52.71 | 8.67E-07 |
| ENSG00000173226 | IQCB1 | SES | IQCB1-201 | -54.89 | 6.11E-03 |
| ENSG00000096746 | HNRNPH3 | SES | HNRNPH3-201 | -58.23 | 1.24E-05 |
| ENSG00000165672 | PRDX3 | A3SS | Ex.TSS.ONT.1965.3 | -60.06 | 3.09E-05 |
| ENSG00000153975 | ZUP1 | SES | ZUP1-202 | -60.25 | 2.61E-03 |
| ENSG00000067365 | METTL22 | SES | METTL22-202 | -61.14 | 6.05E-05 |
| ENSG00000138074 | SLC5A6 | SES | SLC5A6-201 | -69.71 | 3.23E-05 |


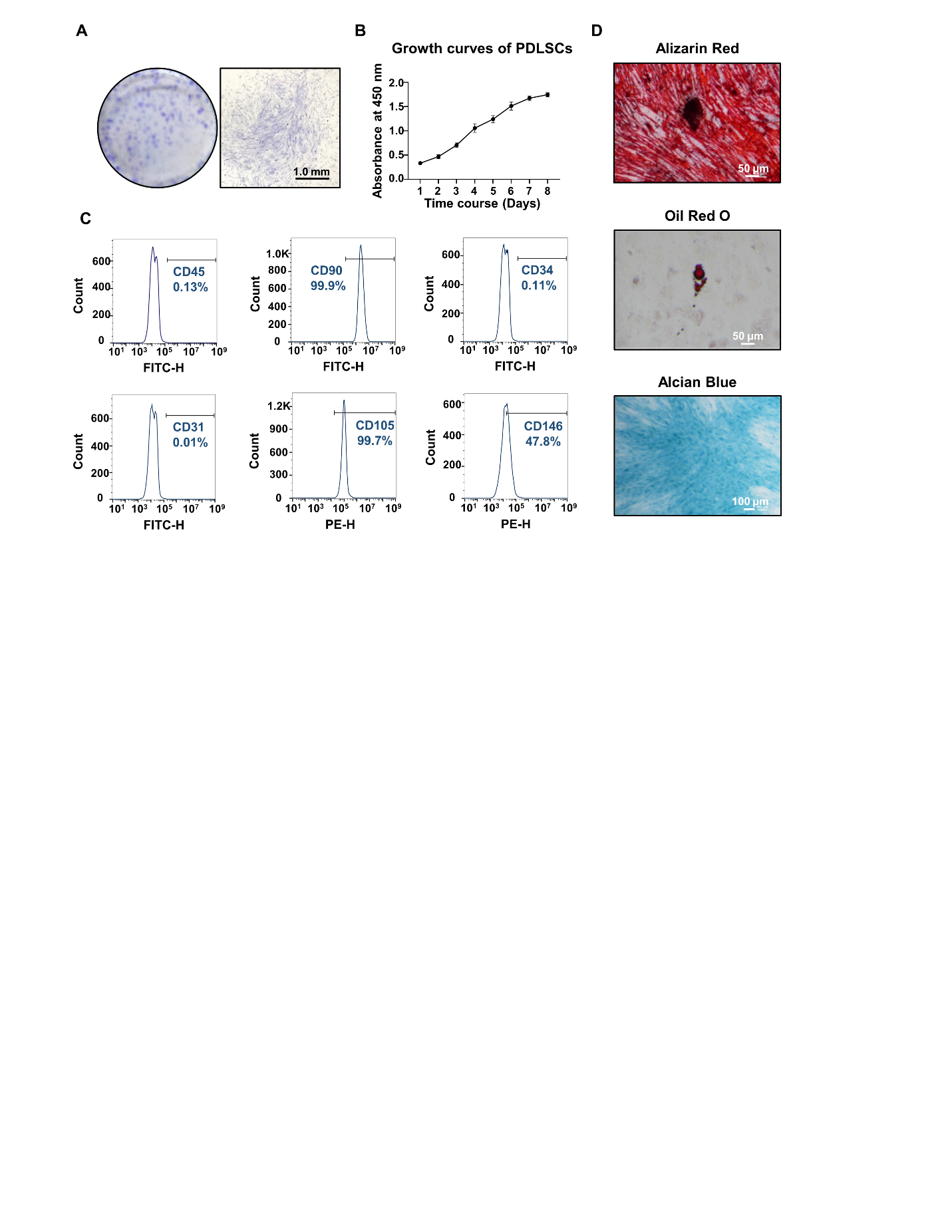


**Figure S1.** Establishment and identification of PDLSCs. **(A)** Global and local images of colony formation by PDLSCs (scale bar: 1.0 mm). **(B)** The proliferation of PDLSCs was determined by a CCK-8 assay. **(C)** The surface markers of PDLSCs were analyzed by flow cytometry. **(D)** PDLSCs were stained with Alizarin red after osteogenic induction (left; scale bar: 50 µm), Oil red O after adipogenic induction (middle; scale bar: 50 µm), and Alcian blue after chondrogenic induction (right; scale bar: 100 µm).


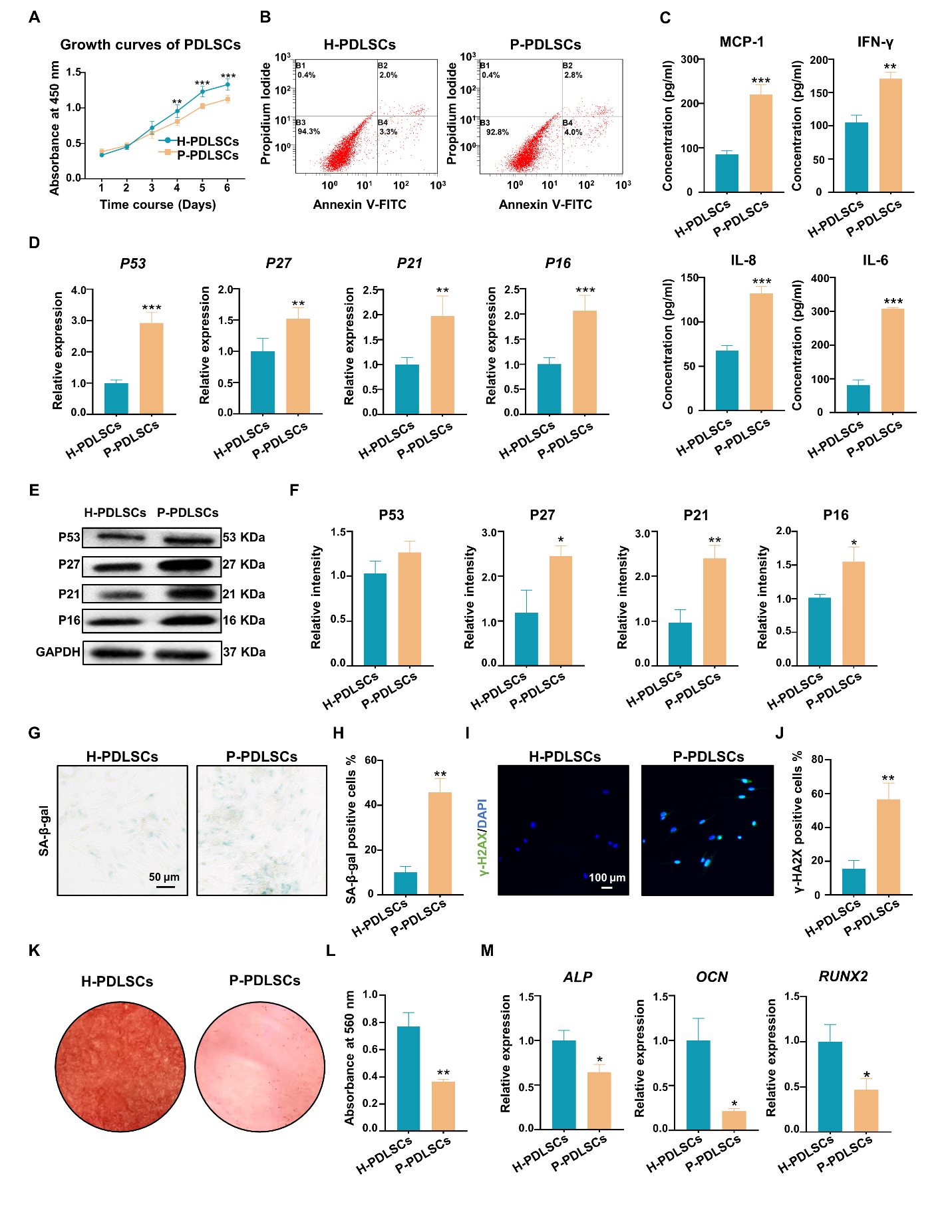


**Figure S2.** P-PDLSCs from the same patient exhibited more senescent characteristics than H-PDLSCs. P-PDLSCs were harvested from teeth affected by periodontitis, and H-PDLSCs were harvested from healthy teeth. **(A)** The proliferation of H-PDLSCs and P-PDLSCs was determined by a CCK-8 assay. **(B)** Apoptosis assay of PDLSCs was analyzed using flow cytometry after staining with Annexin V-FITC and PI. **(C)** The secretion of inflammatory cytokines (MCP-1, IL-8, IL-6, and IFN-γ) into the supernatants of H-PDLSCs and P-PDLSCs was measured via ELISA. **(D)** Expression levels of senescence-related genes (*P53*, *P27*, *P21* and *P16*) in PDLSCs were measured via qRT‒PCR. **(E)** The expression of senescence-related proteins (P53, P27, P21, and P16) was measured via western blotting. All full-length blots are presented in Additional file 2: Fig. S2E. **(F)** Semiquantitative analysis of protein expression levels normalized to GAPDH expression levels. **(G)** Senescence-associated β-galactosidase was analyzed via SA-β-gal staining (scale bar: 50 μm). **(H)** Quantification of SA-β-Gal staining in PDLSCs. **(I)** Immunofluorescence staining for γ-H2AX in PDLSCs; nuclei were stained with DAPI (scale bar: 100 μm). **(J)** Percentage of γ-H2AX-positive cells. **(K)** Representative image of Alizarin red staining of PDLSCs after 28 days of osteogenic induction. **(L)** Quantitative analysis of the calcium mineral deposits formed by PDLSCs after osteogenic induction. **(M)** The expression levels of the osteogenesis-related genes *ALP*, *OCN,* and *RUNX2* in PDLSCs after osteogenic induction were measured via qRT‒PCR. The data are presented as the means ± SDs (n = 3). **p* < 0.05, ***p* < 0.01, and ****p* < 0.001 represent significant differences between the indicated columns.


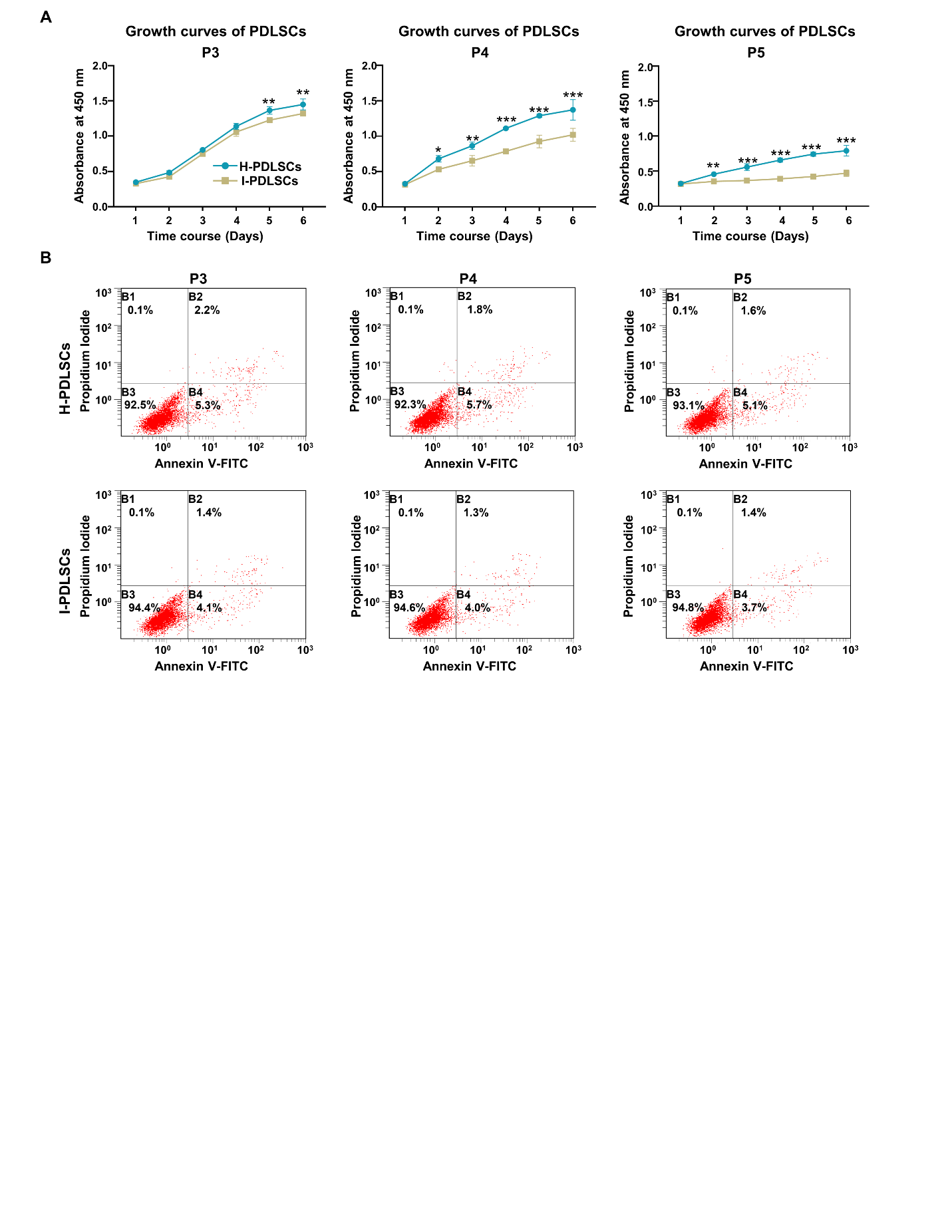


**Figure S3.** I-PDLSCs exhibited more senescent characteristics than H-PDLSCs. **(A)** The proliferation of H-PDLSCs and I-PDLSCs was determined by a CCK-8 assay. **(B)** Apoptosis of H-PDLSCs and I-PDLSCs was determined using flow cytometry. The data are presented as the means ± SDs (n = 3). **p* < 0.05, ***p* < 0.01, and ****p* < 0.001 represent significant differences between the indicated columns.


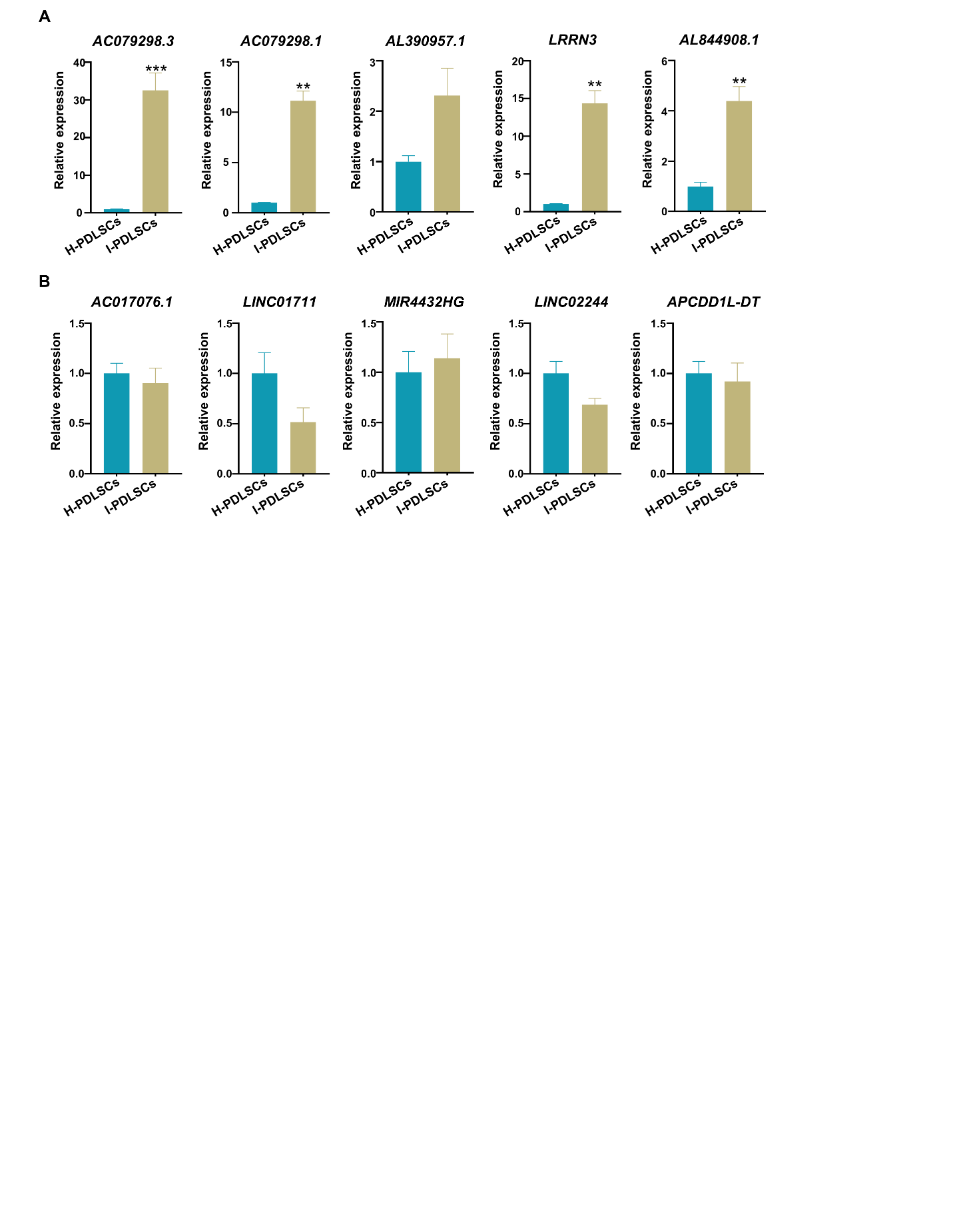


**Figure S4.** Screened lncRNAs associated with PDLSC senescence were identified via microarray analysis **(A-B)** The expression of selected upregulated **(A)** and downregulated **(B)** lncRNAs was verified via qRT‒PCR. The data are presented as the means ± SDs (n = 3). ***p* < 0.01, and ****p* < 0.001 represent significant differences between the indicated columns.


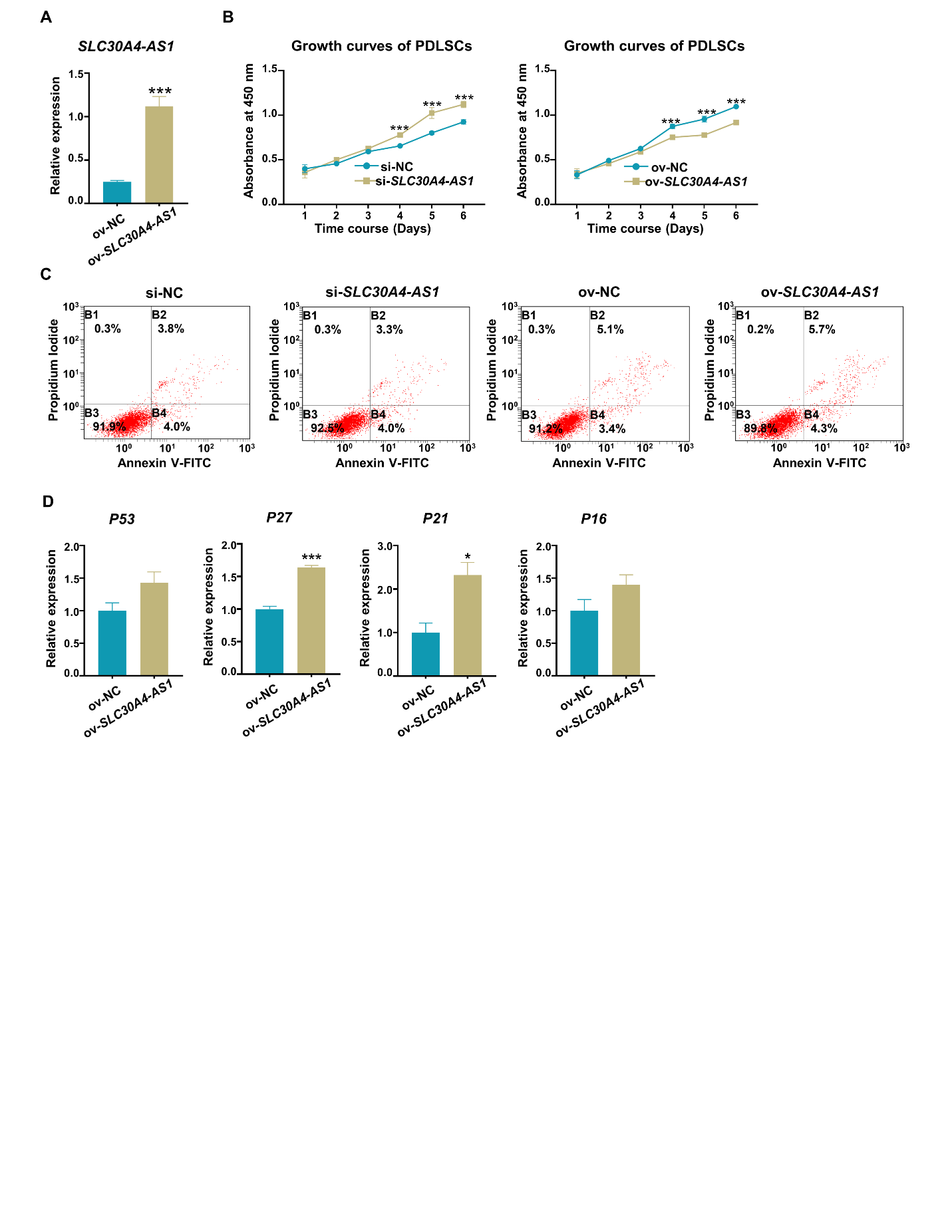


**Figure S5.** *SLC30A4-AS1* regulates the senescence of PDLSCs. **(A)** The efficiency of *SLC30A4-AS1* overexpression in PDLSCs was determined via qRT‒PCR. **(B)** The proliferation of *SLC30A4-AS1*-knockdown (left) or *SLC30A4-AS1*-overexpressing (right) PDLSCs was determined via a CCK-8 assay. **(C)** Apoptosis assay of PDLSCs was analyzed using flow cytometry after staining with Annexin V-FITC and PI. **(D)** Expression levels of senescence-related genes (*P53*, *P27*, *P21* and *P16*) in *SLC30A4-AS1*-overexpressing PDLSCs were measured via qRT‒PCR. The data are presented as the means ± SDs (n = 3). **p* < 0.05, and ****p* < 0.001 represent significant differences between the indicated columns.

**
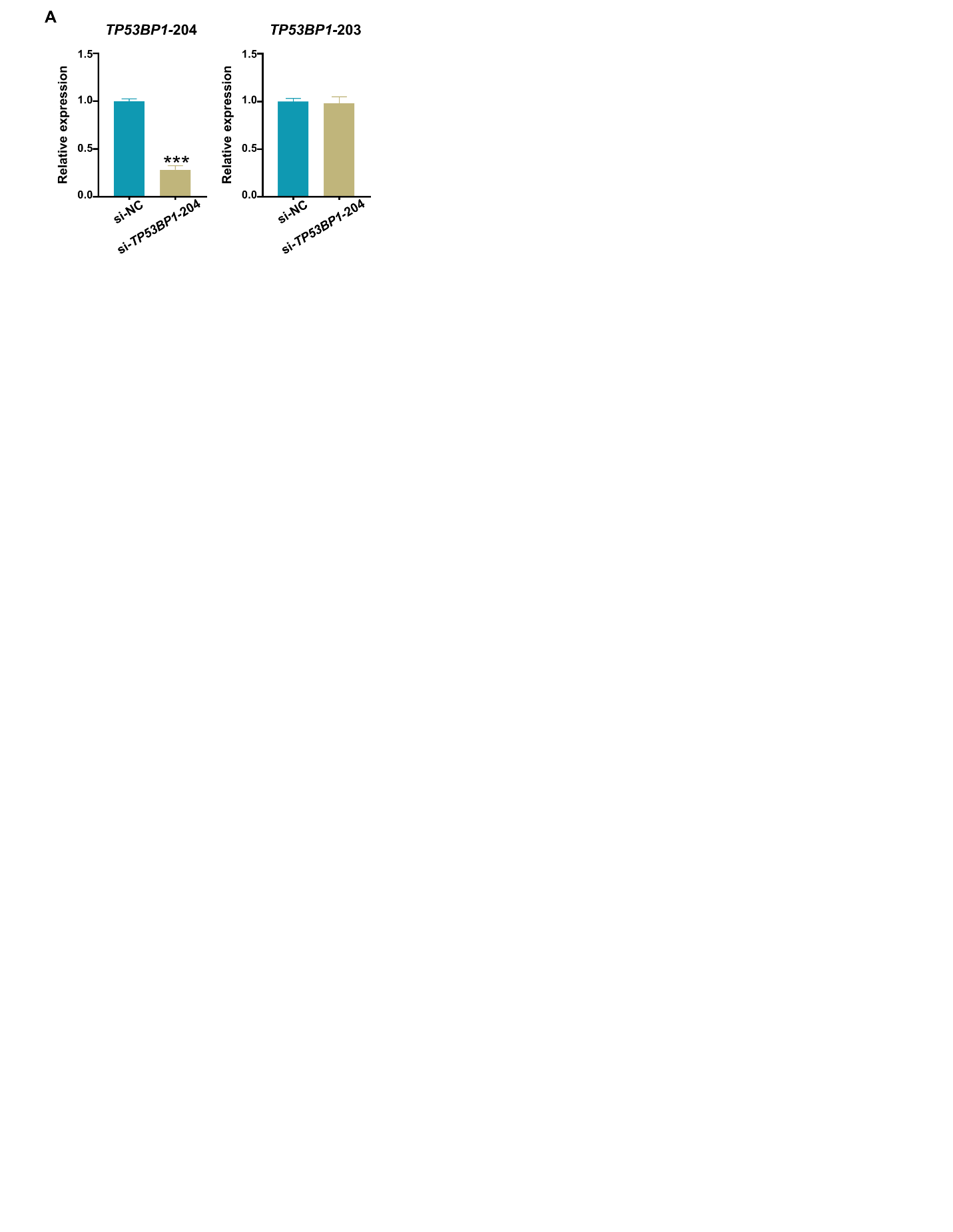
**

**Figure S6.** Knockdown of *TP53BP1*-204 did not affect the expression of *TP53BP1*-203. **(A)** The expression levels of *TP53BP1-*204 (left) and *TP53BP1-*203 (right) in PDLSCs transfected with si-*TP53BP1*-204 were measured via qRT‒PCR. The data are presented as the means ± SDs (n = 3). ****p* < 0.001 represent significant differences between the indicated columns.


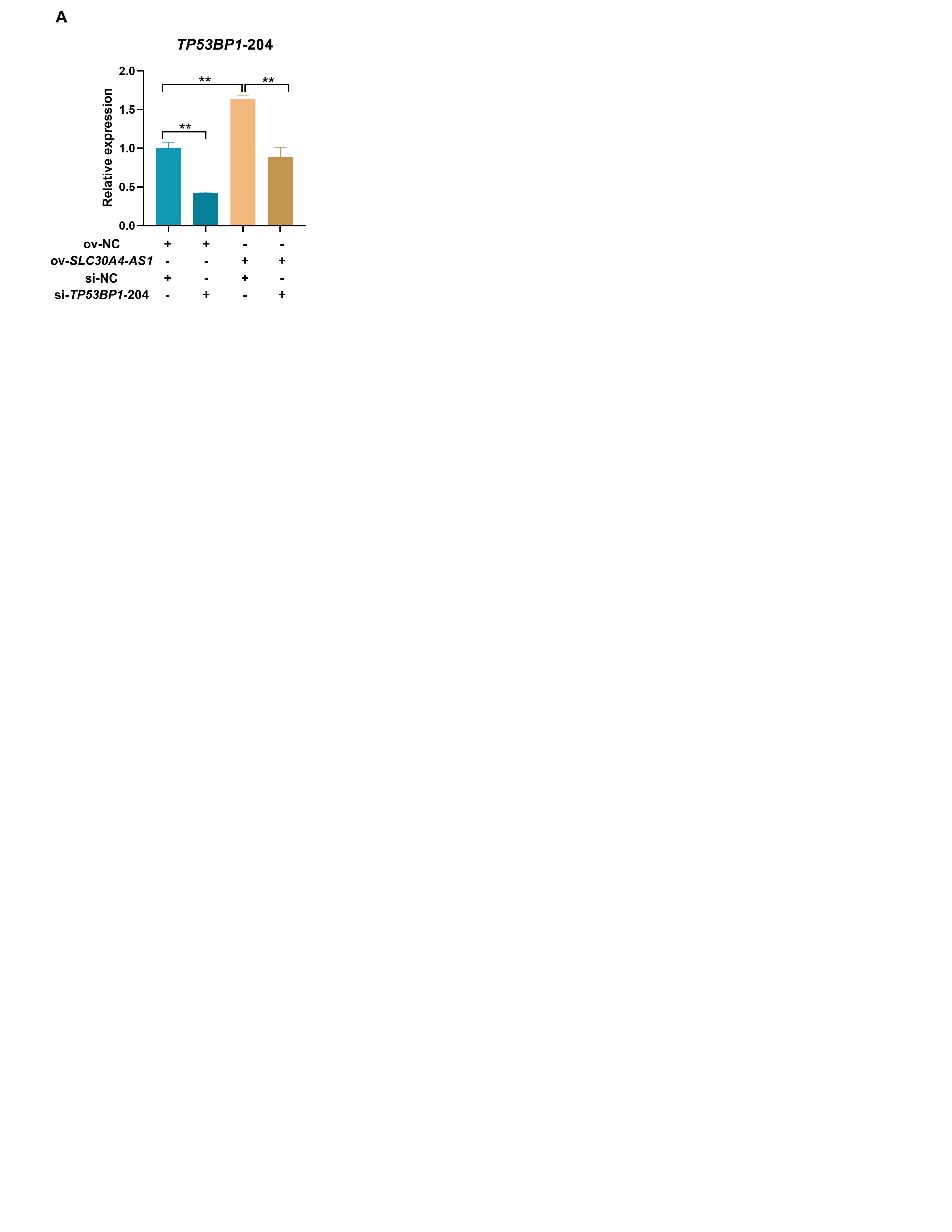


**Figure S7. (A)** Expression levels of *TP53BP1-*204 in PDLSCs transfected with si-*TP53BP1*-204 and ov-*SLC30A4-AS1* were measured via qRT‒PCR. The data are presented as the means ± SDs (n = 3). ***p* < 0.01 represent significant differences between the indicated columns.
